# Susceptibility of *Toxoplasma gondii* to autophagy in human cells relies on multiple interacting parasite loci

**DOI:** 10.1128/mbio.02595-23

**Published:** 2023-12-14

**Authors:** Nicholas Rinkenberger, Alex Rosenberg, Joshua B. Radke, Jaya Bhushan, Tadakimi Tomita, Louis M. Weiss, L. David Sibley

**Affiliations:** 1 Department of Molecular Microbiology, Washington University School of Medicine, St Louis, Missouri, USA; 2 Department of Pathology, Albert Einstein College of Medicine, Bronx, New York, USA; 3 Department of Medicine, Albert Einstein College of Medicine, Bronx, New York, USA; Stanford University, Stanford, California, USA

**Keywords:** intracellular parasite, genetic mapping, quantitative trait locus, linkage analysis, secretory proteins, dense granule proteins, parasitophorous vacuole, interferon, innate immunity

## Abstract

Autophagy contributes to innate immunity by targeting intracellular pathogens for elimination. Previous studies identified a non-canonical autophagy pathway that controls *Toxoplasma gondii* infection in a strain-dependent manner in interferon gamma-activated human cells. Ubiquitination of unknown targets recruits adaptors and LC3 to the parasitophorous vacuole, leading to membrane envelopment and stunted growth. Vacuoles containing strain types II and III are susceptible, while type I strains of *T. gondii* are largely resistant and hence avoid autophagy-mediated growth restriction. Here we interrogated the genetic differences in LC3 recruitment between a resistant type I (GT1) strain and a susceptible type III (CTG) strain of *T. gondii*. We took advantage of a previous genetic cross between these two strains to determine the LC3 recruitment phenotype of 34 unique progeny clones. Genetic linkage mapping revealed that LC3 recruitment was highly multigenic, depending on two major quantitative trait loci (QTLs) on chromosome II and VIII, as well as three minor contributing loci. Ubiquitin affinity capture followed by mass spectrometry identified several potential targets exposed at the surface of the parasitophorous vacuole, including several candidates within the major QTLs. We tested several candidates and identified the dense granule proteins MAF1 on chromosome II, and MAG1 and PSD1 on chromosome VIII, as being partially responsible for susceptibility to LC3 recruitment. Differential susceptibility is likely due to strain-specific differences in recognition of parasite molecules, rather than actively blocking recognition, thus revealing a new mechanism for cell-autonomous restriction of intracellular pathogens.

**IMPORTANCE:**

Autophagy is a process used by cells to recycle organelles and macromolecules and to eliminate intracellular pathogens. Previous studies have shown that some stains of *Toxoplasma gondii* are resistant to autophagy-dependent growth restriction, while others are highly susceptible. Although it is known that autophagy-mediated control requires activation by interferon gamma, the basis for why parasite strains differ in their susceptibility is unknown. Our findings indicate that susceptibility involves at least five unlinked parasite genes on different chromosomes, including several secretory proteins targeted to the parasite-containing vacuole and exposed to the host cell cytosol. Our findings reveal that susceptibility to autophagy-mediated growth restriction relies on differential recognition of parasite proteins exposed at the host-pathogen interface, thus identifying a new mechanism for cell-autonomous control of intracellular pathogens.

## INTRODUCTION

*Toxoplasma gondii* is an obligate intracellular parasite that is able to enter and survive in virtually all forms of nucleated cells from warm-blooded hosts (1). Host cell invasion is an active process that invaginates the host membrane to form a protective niche, called the parasitophorous vacuole (PV), which largely resists fusion with the endomembrane system and supports parasite growth (2). During host cell attachment and invasion, *T. gondii* secretes proteins in three waves from the micronemes (MICs), rhoptries (ROPs), and dense granules (GRAs), respectively (3). Although MIC proteins are primarily involved in attachment to host cells (4), they have also been implicated in signaling through host epidermal growth factor receptor to block autophagy (ATG) and thereby promote parasite survival (5). ROP proteins are secreted directly into the host cell cytosol during invasion and subsequently targeted either to the parasitophorous vacuole membrane (PVM), where they are involved in defending the vacuole against immune effectors or trafficked to the host cell nucleus, where they modulate gene expression (6). GRA proteins are secreted from within the vacuole and either occupy the lumen and decorate the intravacuolar network (7) or are targeted outside the vacuole into the host cell, where they affect signaling or host cell transcription (8). GRA proteins that are exported beyond the PVM are intrinsically disordered and often contain internal repeats that may participate in binding to host cell targets (9). The process of export of GRA proteins beyond the PVM involves several steps including proteolytic processing in the Golgi and translocation across the PVM. The aspartic protease ASP5 resides in the Golgi, where it recognizes a processing sequence (TEXEL motif) in a subset of proteins destined for export from the parasite (10–12). Loss of ASP5 affects localization of both GRA proteins that reside in the lumen of the PVM and those exported beyond the vacuole membrane (13). Comparison of proteins exported in an ASP5- dependent manner (14) reveals that all known GRA effectors are dependent on ASP5, even if they do not contain a conserved TEXEL motif. Subsequently, GRA proteins that are exported beyond the PVM traffic through the MYR1 complex, consisting of at least four separate proteins that insert in the PVM (15, 16). In addition, resident PVM proteins that are exposed on the surface of the vacuole are typically not MYR1 dependent, as shown for GRA15 that activates nuclear factor kappa B (NFκB) in macrophages (17) and interacts with TRAF2/6 to increase clearance in interferon gamma (IFN-γ)-treated human foreskin fibroblast (HFF) cells (18) and MAF1b, which tethers host mitochondria to the surface of the PVM (19, 20). Notably, the *MAF1* locus also contains paralogs known as MAF1a, which also interact with mitochondria but are not required for the recruitment phenotype (21).

Whole genome sequencing of >60 strains of *T. gondii* reveals that they comprise six major clades that share a core genome and differ primarily in families of variable genes that encode secreted pathogenesis determinants (22). In North America and Europe, the population structure is dominated by three clonal lineages referred to as types I, II, and III (23). Although type I strains are relatively rare, they present with greater frequency in some cohorts of susceptible patients (24). In contrast, type II strains are highly prevalent in humans, where they cause infections with variable severity, while type III strains are common in animals but rare in humans (25). These lineages differ substantially in pathogenesis in laboratory mice, with type I being highly virulent, type II being intermediate, and type III being avirulent (26). Previous genetic crosses between these lineages have shown that these differences in acute virulence in the mouse are mediated by differences in secreted effector proteins, notably ROP5, ROP16, ROP17, and ROP18 (26). Importantly, these genes seem less important in mediating pathogenesis in human cells, at least as measured by control of parasite growth in response to IFN-γ in HFF cells (27).

Although naïve cells are highly susceptible to infection, once activated by IFN-γ, they restrict parasite growth though the concerted action of interferon-stimulated genes (ISGs) (28, 29). Human and mouse cells use different mechanisms to restrict parasite growth, although both systems rely on IFN-γ to activate responses. In mice, an expanded family of immunity-related GTPases (IRGs) is upregulated in response to interferon and recruited to the PVM, resulting in vesiculation of the membrane and destruction of the parasite (30). This process is aided by a second family of interferon-inducible, guanylate-binding proteins (GBPs) that are also recruited to the PVM, where they contribute to parasite destruction (31, 32). Importantly, the recruitment of IRGs and GBPs to PVMs in IFN-γ-activated murine cells is highly dependent on a core complex of ATG proteins, including ATG5-ATG12-ATG16 that mediate lipid conjugation of LC3 (33, 34).

Human cells largely lack IRGs (30), yet they rely on select members of the GBP family to restrict parasite growth (35–38). Similar to the murine system, an ATG5-dependent, non-canonical pathway is required for growth restriction of susceptible *T. gondii* strains in human HeLa cells (39). The process initiates with ubiquitination of unknown targets on the PVM, followed by recruitment of ubiquitin-binding proteins (e.g., p62 and NDP52), and finally by decoration with LC3, and engulfment of the PVM by multiple layers of membranes, resulting in a stunted growth phenotype (39). A similar process of ATG-dependent growth restriction has been described in lung A549 cells (40) and in umbilical vein endothelial cells, although in the latter cells, it culminates in delivery to lysosomes (41). The connection between the ATG pathway and IFN-γ is partially mediated by ISG15, which connects ATG proteins with several ISGs in activated human A549 cells (40). Interestingly, there are strain-specific differences in susceptibility to ATG-dependent IFN-γ growth restriction: type I parasites (GT1) are largely resistant to this pathway, while type II (ME49) and type III (CTG) parasites are susceptible (39). Importantly, not all parasites within the cell are affected by the pathway; rather, growth inhibition is only seen in ubiquitin-positive compartments, suggesting that susceptibility is due to recognition of components on the PVM (39). Existing pathogenesis determinants in *T. gondii* do not appear to explain this phenotype, and the molecular basis for recognition of susceptible strains and evasion by resistant strains of the parasite is unknown.

Here, we returned to a previously described genetic cross between type I and III strains and used quantitative trait locus (QTL) mapping (42) to identify loci in the parasite that mediate susceptibility to LC3 recruitment. Our findings reveal two major and three minor interacting loci that collectively control this trait. The QTLs do not contain previously recognized pathogenesis determinants but harbor several candidates that rely on transport machinery previously shown to be essential for export of virulence determinants. Identification of several dense granule proteins that contribute to susceptibility to LC3 recruitment supports a mechanism of differential recognition, rather than active inhibition, in mediating susceptibility to ATG-mediated growth restriction.

## RESULTS

### Susceptibility to LC3 recruitment in IFN-γ-treated cells is strain dependent

**Fig 1:**
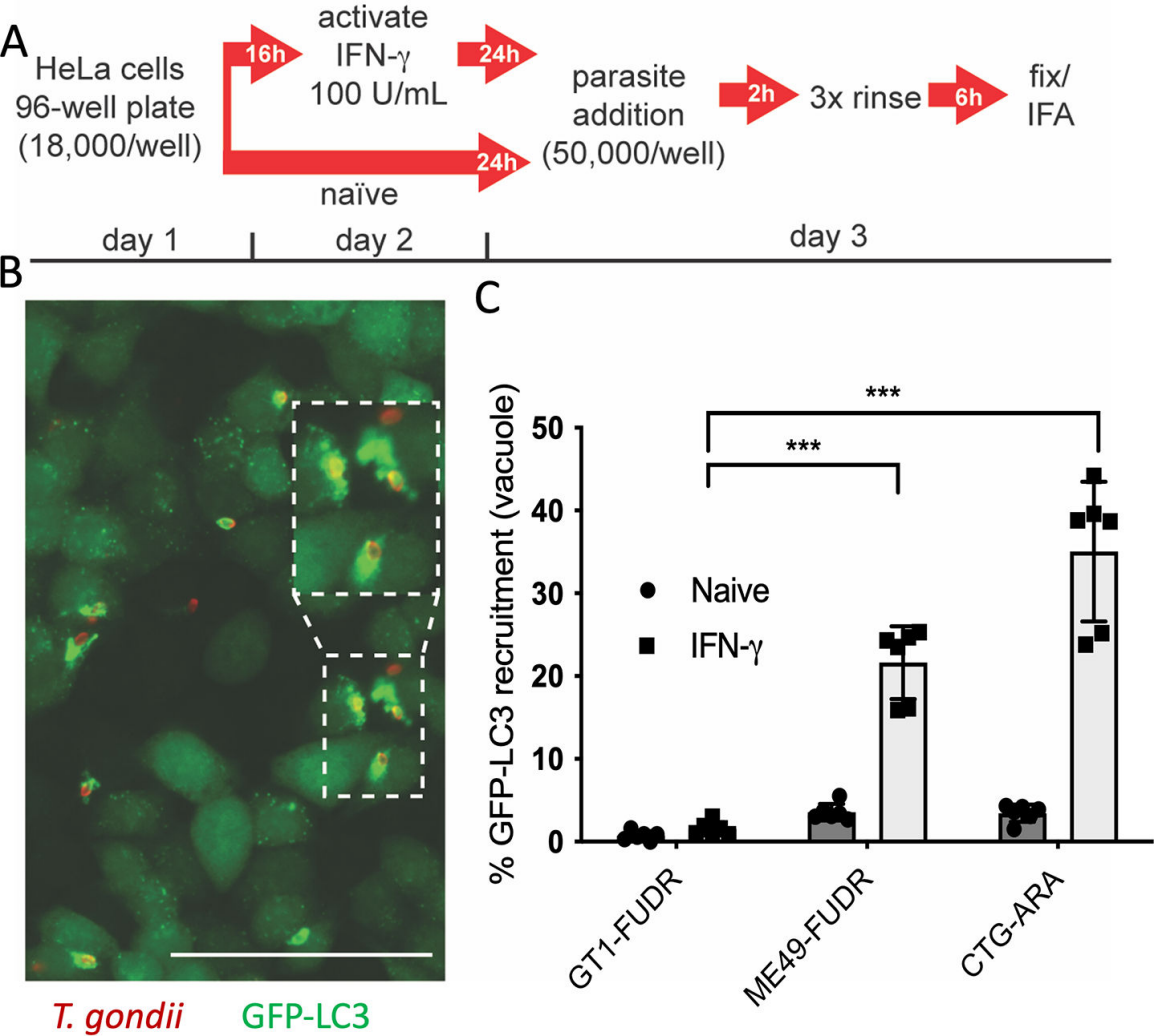
Recruitment of ATG effector LC3 to the PVM is strain dependent and requires INF-γ. (**A**) Schematic of GFP-LC3 recruitment assay to the parasitophorous vacuole membrane (PVM). HeLa cells were inoculated into 96-well plates and allowed to adhere overnight, then activated for 24 h with IFN-γ (100 U/mL) or left untreated prior to the addition of parasites (50,000/well). Parasites were allowed to invade for 2 h; wells were rinsed three times and replaced with fresh media before fixation at 6 h post-infection (hpi). (Β) Example image of GFP-LC3-positive recruitment to the PVM. GFP-LC3 (green perimeter) localized to the PVM surrounding CTG-ARA strain parasite (red) in IFN-γ (100 U/mL)-stimulated cells. Parasites were identified with a rabbit polyclonal anti-RH antibody (red) and GFP-LC3 localized with a mouse anti-GFP (green) antibody. Images were collected at ×20 magnification using a Cytation 3 multimode plate imager. Inset shows an enlarged example. Scale bar = 50 μm. (**C**) Quantification of strain-specific differences of GFP-LC3 recruitment to the PVM using immunofluorescence imaging in naïve (solid circles) and IFN-γ-activated HeLa cells (100 U/mL, solid squares) for type I (GT1-FUDR), type II (ME49-FUDR), and type III (CTG-ARA) strain parasites. Recruitment percentages were calculated as (GFP positive vacuoles / total vacuoles) × 100 for each strain and indicated condition. Means ± standard error of the mean for three independent experiments, each with internal technical duplicates. Statistical comparison: ***P ≤ 0.005, two-way analysis of variance, Tukey’s multiple comparisons test.

Previous image-based approaches to evaluate host effector recruitment to the PVM involved low-throughput, immunofluorescence-based assays whereby multiple host effector proteins were evaluated individually to enumerate positive recruitment events on the PVM. To expand our ability to evaluate multiple strains and conditions simultaneously, we adapted a high-throughput, imaged-based screen similar to that previously described in studies of small molecule inhibitors (43, 44). We evaluated recruitment of green fluorescent protein fused to light chain 3 (GFP-LC3) to the PVM surrounding parasites in naïve and IFN-γ-stimulated HeLa cells (Fig. 1A). We chose LC3 recruitment as a readout because it is one of the final steps leading to growth inhibition in IFN-γ-activated human cells (39, 40). Recruitment of LC3 to the PV was quantified based on signal overlap of GFP-LC3 (green) and *T. gondii* parasites (red) (Fig. 1B, example of positive recruitment event inset) using an automated analysis of images collected from 96-well plates using a Cytation 3 multimode plate reader. First, we evaluated the parental strains used in previous genetic crosses (GT1-FUDR [type I], ME49-FUDR [type II], and CTG-ARA [type III]) (45–48) to determine their susceptibility and develop scoring criteria for the high-content assay. As previously observed, recruitment of LC3 to the PVM was rare in naïve cells, regardless of the genetic background of the parasite (Fig. 1C). However, in IFN-γ-activated cells (100 U/mL), type I (GT1-FUDR) parasites were resistant to GFP-LC3 recruitment, while type II (ME49-FUDR) and type III (CTG-ARA) strains were significantly more susceptible to GFP-LC3 recruitment (Fig. 1C). The resistant mutants on different genetic backgrounds behave very similarly to previous reports using wild-type strains (39, 40), indicating that they provide a reliable indicator of genetic susceptibility. Based on these phenotypic differences, we selected the type I × type III genetic cross (45, 46) to map the observed strain-dependent differences in ATG effector recruitment.

### Progeny of type I × type III cross has mixed LC3 recruitment phenotypes

**Fig 2:**
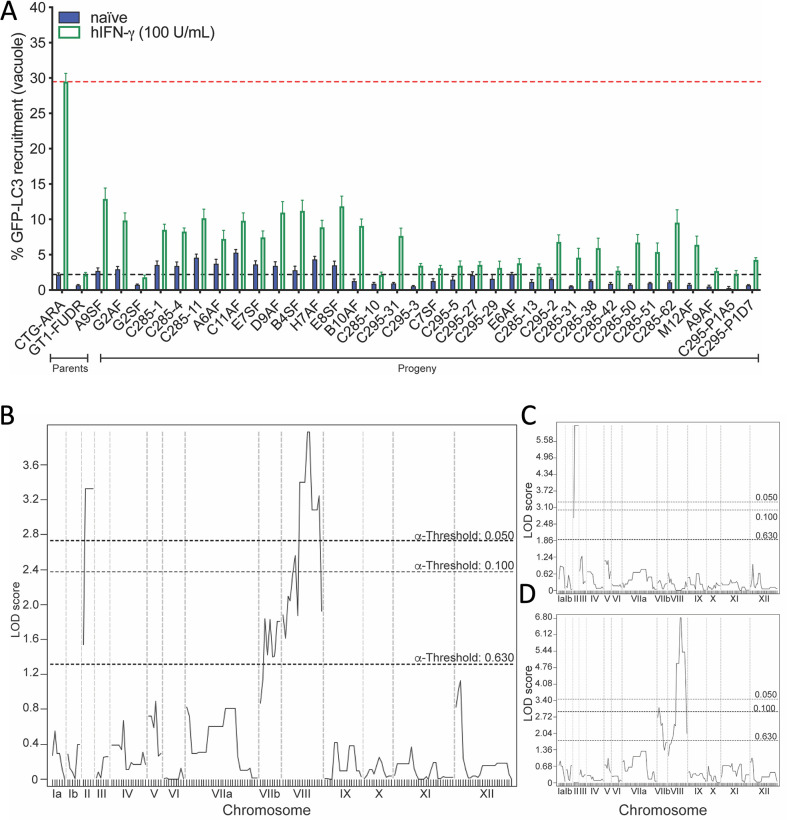
Evaluation of susceptibility of I x III progeny to autophagy effector recruitment. (**A**) Quantification of GFP-LC3 recruitment for all 34 F1 progeny of the I × III genetic cross in naïve (blue bars) or IFN-γ-activated HeLa cells (100 U/mL, green outline bars). GFP-LC3 expressing HeLa cells were plated in 96-well plates, allowed to adhere overnight before stimulation with IFN-γ (100 U/mL), or left unstimulated (see Fig. 1A). Parasites (5 × 10^4^) were allowed to invade for 2 h; wells were rinsed three times and replaced with standard culture media and cultured for 6 hpi prior to fixation and staining. Images were collected using a Cytation 3 multimode plate imager at ×20 magnification (see Fig. 1B). Percent GFP-LC3 recruitment was calculated by (GFP-LC3-positive vacuoles / total vacuoles) × 100 for all conditions and strains. Values provided as mean ± standard error of the mean of at least three biological replicates with internal duplicates. The black dotted line denotes percent recruitment for GT1-FUDR (type I parent) in IFN-γ-activated HeLa cells. The red dotted line denotes percent recruitment for CTG-ARA (type III parent) in IFN-γ activated HeLa cells. (**B**) Single scan QTL mapping of GFP-LC3 recruitment identified two major peaks, one on chromosome ChrII (logarithm of odds [LOD] 3.325, *P* = 0.017) and a second on ChrVIII (LOD 3.974, *P* = 0.007). Indicated LOD score thresholds are based on 1,000 random permutations. (**C**) Single-genome scan with ChrVIII as a covariate enhanced the QTL peak on ChrII (LOD 6.169, *P* = 0.002). (**D**) Single-genome scan with ChrII as a covariate identified two peaks on ChrVIII (LOD 6.818, *P* = 0.000; LOD 3.117, *P* = 0.080). Chromosomes are labeled on the *x*-axis, and boundaries are marked by gray dotted lines. Black dotted lines indicate increasing confidence presented as α-thresholds (0.630, 0.100, and 0.050). For genetic marker details and LOD scores, see Tables 1 and S4.

In order to identify the *T. gondii* effector(s) responsible for mediating strain-specific differences in host ATG response to infection in human cells, we quantified GFP-LC3 recruitment phenotypes of 34 unique F1 progeny isolated from the type I × type III genetic cross (45, 46) using the image-based recruitment assay described in Fig. 1 (data listed in Table S1). Recruitment of GFP-LC3 in naïve HeLa cells was low for all progeny, although there was an interesting trend that clones with high levels of recruitment in naïve cells were also the highest when treated with IFN-γ (Fig. 2A). Irrespective of the basal level, all progeny exhibited an IFN-γ-dependent increase in GFP-LC3 recruitment to the PVM (Fig. 2A). Interestingly, none of the progeny were categorized as highly susceptible (none exceeded ~13% GFP-LC3-positive vacuoles) nor did they reach the level seen for the type III parent (CTG-ARA, Fig. 2). Twenty progeny clones were categorized as having intermediate susceptibility, where 5%–13% of PVMs were GFP-LC3 positive in the presence of IFN-γ (Fig. 2A). Fourteen of the progeny clones were resistant to ATG effector recruitment (<5% GFP-LC3-positive vacuoles) and closely phenocopied the type I parent (GT1-FUDR, Fig. 2A). Taken together, these data reinforce differences in susceptibility of *T. gondii* strains to IFN-γ-mediated recruitment of LC3 to PVMs and demonstrate that it is a heritable genetic trait that is likely mediated by multiple loci.

### Quantitative trait locus mapping identifies two interacting loci control LC3 recruitment

To identify genetic loci responsible for the IFN-γ-dependent, strain-specific differences we performed a single locus scan, genome-wide association analysis of the LC3 recruitment phenotypes of 34 F1 progeny using J/qtl (49). The single-genome scan identified two significant QTLs, one peak on chromosome II (ChrII) (3.32 logarithm of odds (LOD) score, marker W35487) and a second on ChrVIII (3.97 LOD, marker AK113), indicating multiple genes are involved in the phenotype (Fig. 2B; Table 1). To determine the impact of each locus, we fixed the ChrVIII locus as a covariate and repeated the scan, resulting in a doubling of the ChrII LOD score (6.169, marker W35487), but no other significant peaks were identified (Fig. 2C; Table S2). When the variance associated with the ChrII locus was fixed as a covariate, the ChrVIII QTL was highly significant (6.81 LOD, marker AK50), and additionally, a second peak was observed on ChrVIIb, though this peak did not meet statistical significance (*P* = 0.08, marker L363) (Fig. 2D; Tables 1 and S2). Combined, the QTLs identified on ChrII (163 genes, spanning 1.29 × 10^6^ bp) and ChrVIII (369 genes, spanning 2.65 × 10^6^ bp) account for 77.4% of the variance observed among the progeny in the LC3 recruitment phenotype (Tables 1 and S2).

**Table:** Table 1 Summary of quantitative trait loci for LC3 recruitment

Chromosome	LOD^*a*^	Markers^*b*^	Genes	95% CI^*c*^	Interaction
II	3.32	L351-W35487-L31-T3	163	1.29e6 bp (1360683–70338)	Additive and two locus QTL with VIII
VIII	3.97	SRS1-AK113-GRA1	369	2.65e6 bp (2660855–5310946)	Additive and two locus QTL with II
VIIb	3.11	L363-AK105	nd	1.26e6 bp (844047–2109662)	II as covariant
VIIa / X	4.03	SAG4 on VIIa L366 on X	nd	292724 peak 6447616 peak	Interactive

^*a*^ LOD, log odd ratio.

^*b*^ Genetic markers from ToxoDB.org.

^*c*^ Genetic size (and position) based on ToxoDB.org.

### Further analysis of interactions reveals multiple contributing loci

**Fig 3:**
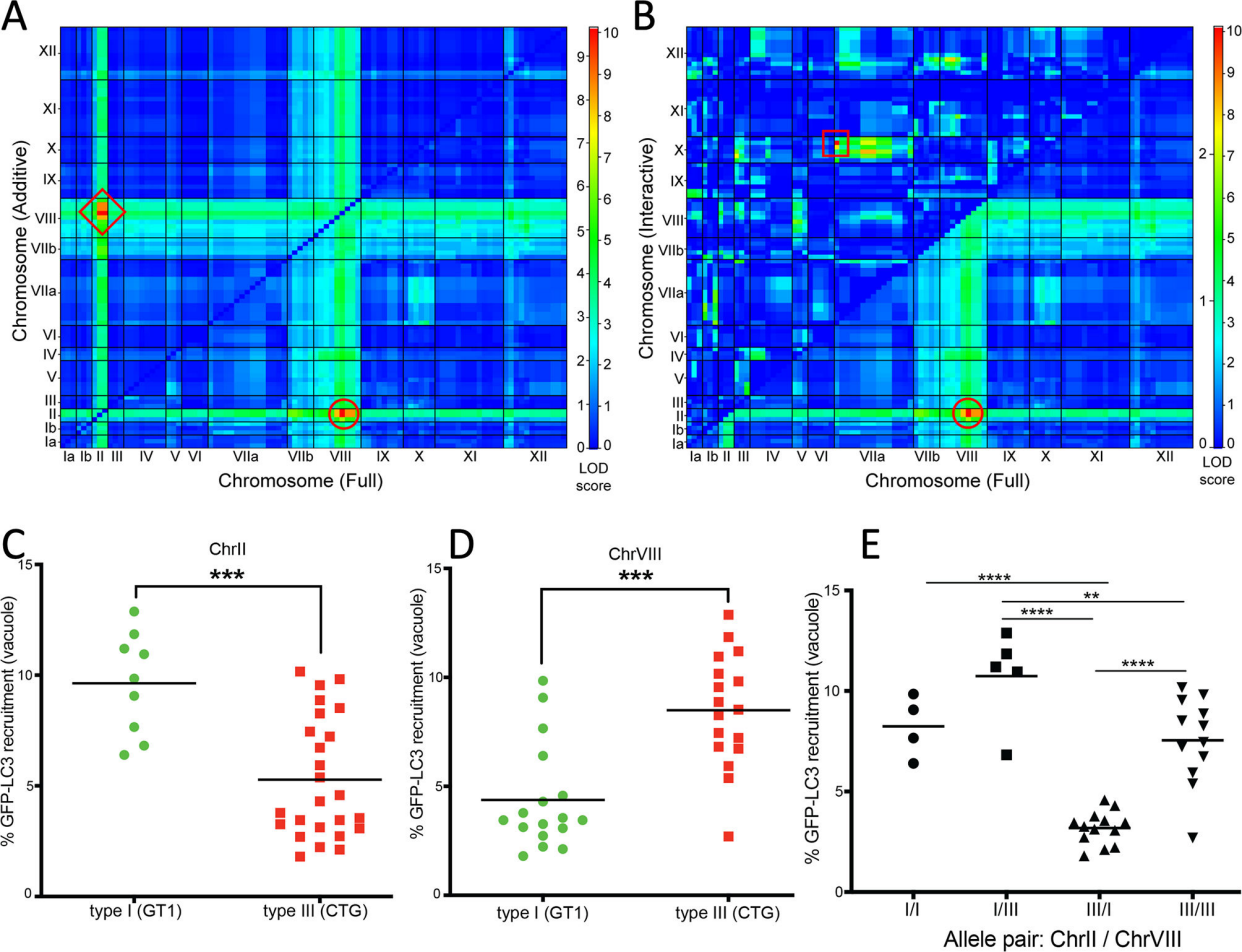
Multiple interacting loci contribute to the LC3 recruitment phenotype. (**A**) Pairwise scan of ATG recruitment of 34 progeny of the type I × type III genetic cross across all 13 chromosomes identified additive interactions between QTLs. The lower right triangle shows the full two-locus QTL with interaction between marker pair KT-L379A/AK113 (ChrII/ChrVIII, 10.14 LOD, *P* = 0.00; red circle). The upper left triangle defines additive interactions between markers L31-T3/AK113 (9.68 LOD *P* = 0.00, red diamond). (**B**) Pairwise scan of ATG recruitment of 34 progenies of the type I × type III genetic cross across all 13 chromosomes identified interactive loci contributing to ATG recruitment. The lower right triangle corresponds to the QTLs described in A with the corresponding red circle to highlight the locus. The upper left triangle identifies interactions between markers SAG4 and L366 (ChrIIa and ChrX, 4.03 LOD, *P* = 0.34, not significant; red square). The color bar to the right of each graph represents LOD scores across all pairwise combinations of markers on all chromosomes, with red colors representative of higher LOD scores and blue colors representative of lower LOD scores. The left side of the color bar defines LOD scores for the upper left triangle, and the right side of the color bar defines LOD scores for the lower right triangle. For genetic marker details and LOD scores, see Table S5. (**C**) Susceptibility to GFP-LC3 recruitment in progeny that inherited the type I allele (green circles) or the type III allele (red squares) from ChrII. Genetic marker L31-T3 is presented, although all RFLP markers within the ChrII QTL have identical allelic inheritance. ***P ≤ 0.0005, Welch’s unpaired *t*-test. (**D**) Susceptibility to GFP-LC3 recruitment in the progeny that inherited the type I allele (green circles) or type III (red squares) on ChrVIII at marker AK113. Two genetic markers define the ChrVIII peak with identical allelic inheritance patterns. *** P ≤ 0.005, Welch’s unpaired *t*-test. (**E**) I and III represent parental alleles inherited by all 34 progeny defined by the main QTLs on ChrII and ChrVIII. Inherited alleles listed in the following order: ChrII/ChrVIII. Black lines indicate mean GFP-LC3 recruitment for all progenies. Data based on median GFP-LC3 recruitment from IFN-γ-stimulated HeLa cells as described in Fig. 2. **P ≤ 0.01, ****P ≤ 0.0001; two-way analysis of variance, Tukey’s multiple comparisons test.

To further define the nature of the interaction between the ChrII and ChrVIII QTLs and to identify other potential interactions, we performed pairwise, two-locus genome scans using several models (50, 51). Such pairwise scans can be used to define the relationships between QTLs, including additive (each peak is independent of the other), non-additive (peaks are dependent on a second locus), and interactive (contribution requires both loci) (49–51). The interaction between marker pairs KT-L379A (ChrII) and AK113 (ChrVIII) was observed in the full QTL plot on the bottom right (10.14 LOD, *P* = 0.00; Fig. 3A, red circle) and in the additive plot on the upper left between marker pairs L31-T3 (ChrII) and AK113 (9.68 LOD, *P* = 0.00; Fig. 3A, red diamond). The additive model was highly significant, suggesting these peaks are independent of each other (Table S3). To determine if there were any interactive effects, we compared the two-locus full scan to an interactive model (Fig. 3B). We identified a new interactive pair of QTLs between ChrVIIa and ChrX defined by markers SAG4 (ChrVIIa) and L366 (ChrX) (Fig. 3B, red square, upper left triangle). This interacting pair has a 4.03 LOD score but does not achieve statistical significance (*P* = 0.34) (Tables 1 and S3). This interaction is mirrored by a second marker, AK132 on ChrX, that also interacts with SAG4 on ChrVIIa at the (1.33 LOD, *P* = 0.99; red square). These paired QTLs on ChrVIIa and ChrX are only seen in the interaction plot (Fig. 3B, upper left) but not in the full QTL scan (Fig. 3B, lower right), indicating they likely depend on each other. Together, these analyses identify five potential QTLs that mediated the GFP-LC3 recruitment phenotype: two additive QTLs on ChrII and ChrVIII, a third minor QTL on ChrVIIb, and two interactive loci on ChrVIIa and ChrX.

### Allelic inheritance at two major QTLs contributes differentially to susceptibility to LC3 recruitment

To determine which alleles inherited by the progeny of the type I × type III cross were driving the genetic susceptibility to LC3 recruitment in human cells, we correlated the % GFP-LC3-positive vacuoles of each F1 progeny with each restriction fragment length polymorphism (RFLP) marker contained within the peak of each major QTL. Surprisingly, susceptibility to LC3 recruitment at the ChrII (Fig. 3C; e.g., marker L31-T3) locus tracked with the type I allele (average 9.63% GFP-LC3-positive vacuoles, green circles), with progeny inheriting the type III allele on ChrII being more resistant to GFP-LC3 recruitment (average 5.28% GFP-LC3-positive vacuoles; red squares) (Fig. 3C). In contrast, allelic inheritance at the ChrVIII (e.g., marker AK113) locus matched the observed phenotype of the parental strains, with susceptible strains harboring the type III allele (average 8.49% GFP-LC3-positive vacuoles; red squares) and resistant strains containing the type I allele (average 4.38% GFP-LC3-positive vacuoles; green circles) (Fig. 3D). Next, we evaluated parental alleles inherited by each progeny to determine the influence of allelic pairs on susceptibility (Fig. 3E). The most susceptible progeny from the cross inherited the type I allele from ChrII and the type III allele from ChrVIII (genotype I/III, average 10.74% GFP-LC3-positive vacuoles). Progeny that inherited the type I allele at both QTLs (genotype I/I, average 8.25% GFP-LC3-positive vacuoles) or the type III allele from both QTLs (genotype III/III, average 7.55% GFP-LC3-positive vacuoles) showed intermediate susceptibility (Fig. 3E). The most resistant progeny to LC3 recruitment inherited the type III allele from ChrII and the type I allele from ChrVIII (genotype III/I, average 3.19% GFP-LC3-positive vacuoles). The allelic combination III/I was significantly different from all other possible allelic combinations (Fig. 3E). Despite the differential contribution of loci, the pattern of inheritance of the type III allele from ChrVIII most closely correlated with susceptibility to IFN-γ-dependent ATG recruitment. The QTL in ChrVIII is defined by the flanking markers SRA1 and GRA1 (95% CI) that together spans 369 genes. Examination of their putative functions based on gene annotations in ToxoDb.org did not identify any previously defined virulence genes, indicating the LC3 susceptibility phenotype is mediated by novel factors.

### Susceptibility to LC3 recruitment requires ASP5 but not MYR1

**Fig 4:**
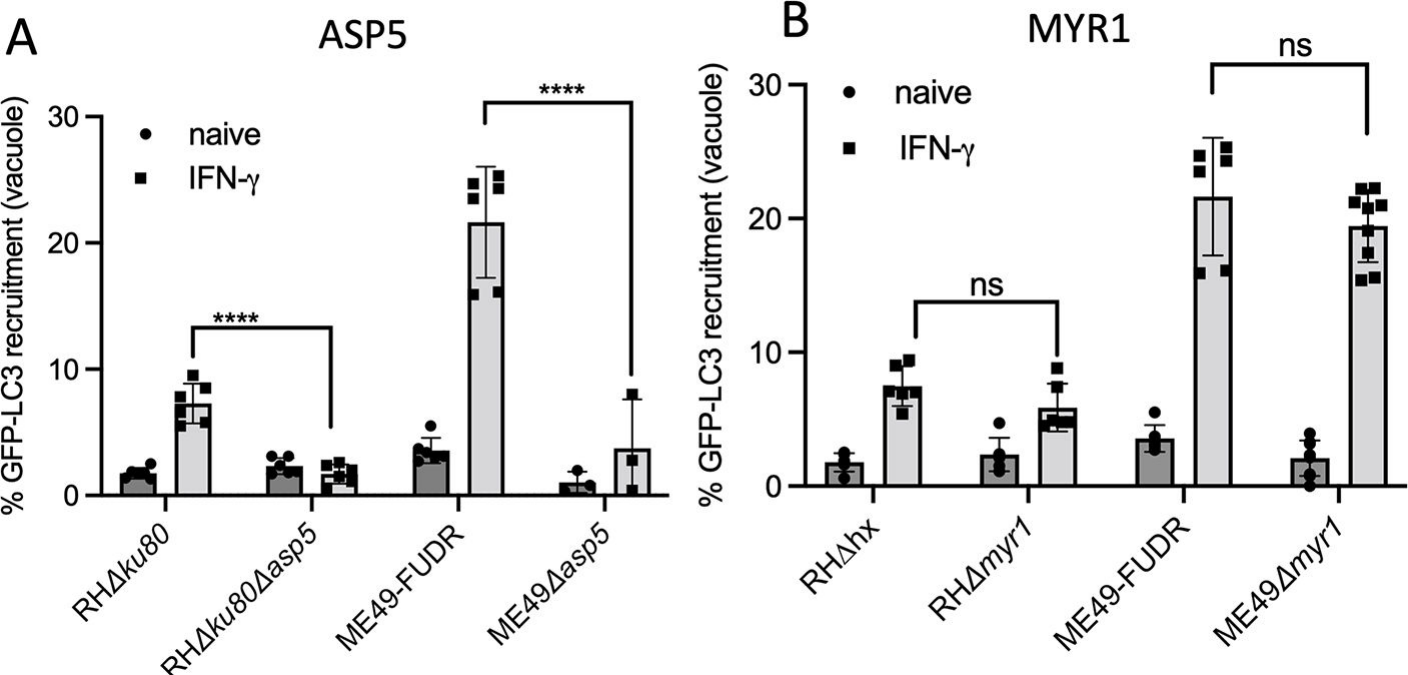
Dependency of LC3 recruitment on ASP5 and MYR1. (**A**) Parental type I (RH∆*ku80*) or type II parasites (ME49-FUDR) were compared to the *∆asp5* knockout strains in their respective genetic backgrounds (type I: RH∆*ku80*∆*asp5* or type II: ME49*∆asp5)*. (**B**) Parental type I (RH∆hxgrpt) or type II parasites (ME49-FUDR, ME49-FLuc) were compared to parasites lacking MYRI (type I: RH∆*myr1* or type II: ME49∆*myr1*). Recruitment percentages were calculated as (GFP positive vacuoles/total vacuoles) × 100 for each strain and indicated condition. Quantification of GFP-LC3 recruitment in naïve (circles) and IFN-γ-activated (100 U/mL) (squares) HeLa cells was evaluated by immunofluorescence imaging using the Cytation 3 multimode plate imager at ×20 magnification. Results presented as mean ± standard error of the mean of at least three biological replicates with two technical replicates each. At least 200 vacuoles were evaluated per replicate. Statistical analysis: ****P ≤ 0.0001; two-way analysis of variance, Tukey’s multiple comparisons test. ns, not significant

ATG-dependent growth restriction results in LC3 deposition on the PVM; hence, we considered it likely that the target(s) that explain differential sensitivity might lie at this interface. Many previously characterized parasite secretory proteins that are trafficked to the PVM or beyond are processed by an aspartic protease in the parasite Golgi called ASP5 (10–12) and then transferred across the PVM by a protein complex known as MYR (15, 16). To assess the role for ASP5 in export of effectors that might alter susceptibility to ATG, we quantified GFP-LC3 recruitment in naïve and IFN-γ-activated HeLa cells in wild-type and knockout parasite lines, taking advantage of previously derived mutants in the type I and II backgrounds. Wild-type strains that express ASP5 followed the expected strain-specific differences in LC3 recruitment, with type I parasites being resistant and type II parasites being susceptible in IFN-γ-activated HeLa cells (Fig. 4A). Surprisingly, in IFN-γ-activated HeLa cells, both type I and type II parasites that lacked ASP5 were more resistant to GFP-LC3 recruitment, and they each showed significant decrease from their wild-type counterparts (Fig. 4A; Table S2). These data suggest a role for ASP5 in processing of parasite effector protein(s) that is subsequently exported to the PVM or beyond and that recognition of this factor influences LC3 recruitment. To determine if susceptibility to LC3 recruitment was dependent on MYR1, we quantified strain-specific differences in recruitment of GFP-LC3 to the PVM in naïve and IFN-γ-activated HeLa cells in wild type and ∆*myr1* mutant parasites. Genetic ablation of MYR1 in type I RH∆*myr1* or type II ME49Δ*myr1* parasites did not alter their LC3 recruitment phenotypes relative to the parental controls (Fig. 4B; Table S3). These findings indicate that the susceptibility of *T. gondii* to LC3 recruitment is mediated by an effector that is ASP5 dependent but not MYR1 dependent.

### Identification of candidate genes in the QTLs

Based on the above phenotypes, we reasoned that resistance versus susceptibility of different *T. gondii* strains to LC3 recruitment is likely mediated by an exported protein that is ASP5 dependent and MYR1 independent. As such, we categorized the genes under the two major QTLs based on whether they showed dependence on ASP5 for export in a previous proteomic study (14). We also scored them for whether they were detected in a MYR1 interactome (52), consistent with being located on the PVM. Finally, we cross indexed these hits with permissive biotin affinity labeling experiments that were conducted with the PVM resident proteins GRA13, GRA17, and GRA23 (53). We then applied additional filtering criteria to identify those with predicted signal peptides, strain-specific polymorphisms, and potential subcellular localization to dense granules or the PV based on LOPIT together with existing data from published studies or annotations (https://toxodb.org). The resulting list includes a number of known secretory proteins as well as many hypothetical proteins (Data set 1). The ChrII QTL contains the *MAF1* locus that contains several copies of related genes *MAF1a* and *MAF1b*, the latter of which is implicated in mitochondrial recruitment (20, 54) (Data set 1). Additionally, near the end of the QTL on ChrVIII is the gene encoding MAG1, which is a dense granule protein that is a prominent component of the cyst matrix (55). MAG1 is also expressed in tachyzoites, where it was recently shown be secreted beyond the PVM and to inhibit interleukin (IL)-1β production (56). MAG1 was identified in the ASP5-dependent proteome and was also present in the GRA17 and GRA25 BioID data sets, as well as the MYR1 interactome (Data set 1). This region on ChrVIII also contains a gene encoding a phosphatidyl serine decarboxylase (PSD), which was previously shown to be secreted from dense granules into the PV and associated with the PVM (57). PSD1 was also identified in the GRA17 and GRA25 BioID data sets as well as the MYR1 interactome (Data set 1).

### Ubiquitin affinity capture of proteins at the host-parasite interface

**Fig 5:**
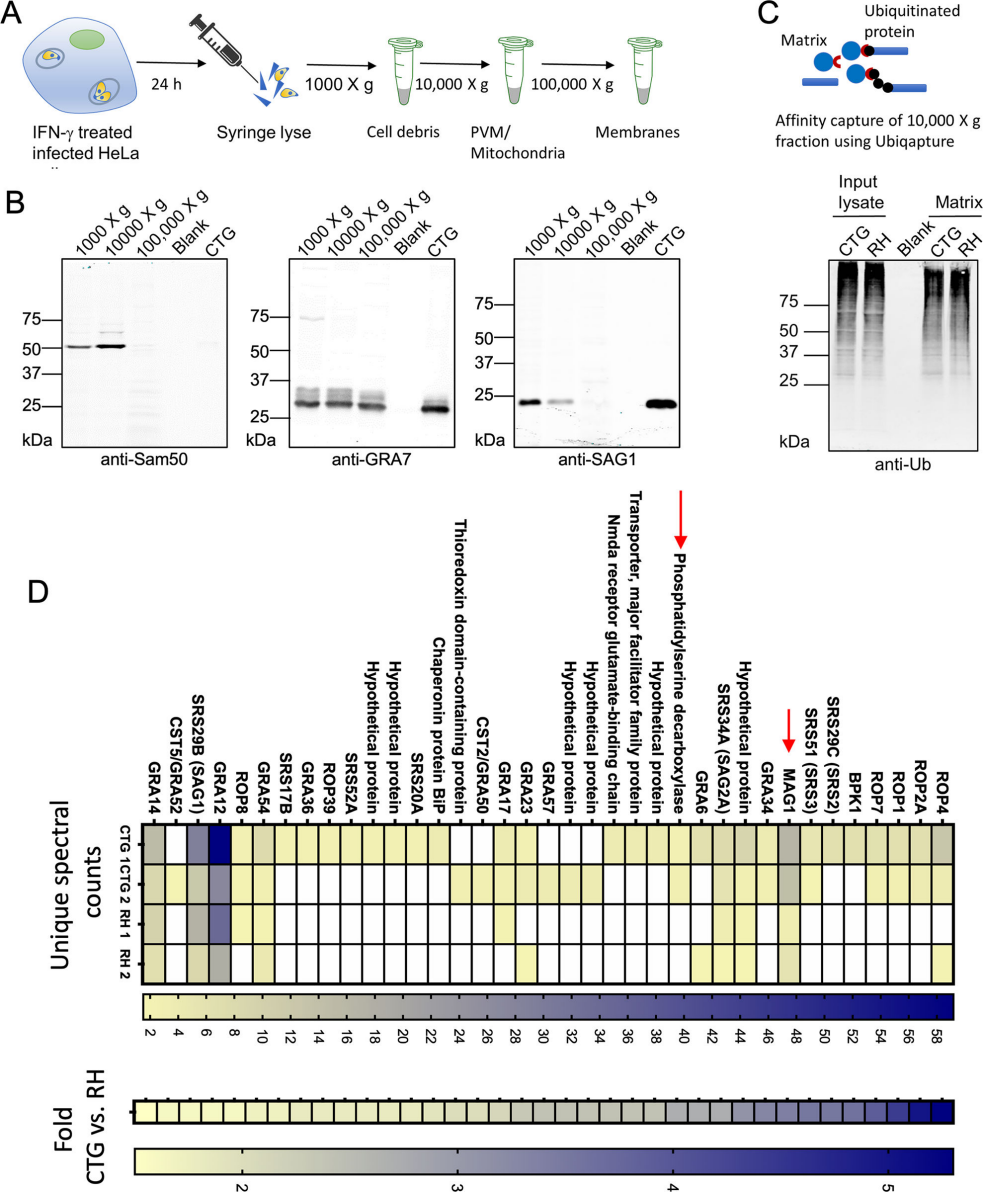
Identification of differentially ubiquitinated parasite proteins. (**A**) Schematic representation showing isolation of PVM enriched fraction from HeLa cells. IFN-γ-treated HeLa cells were infected with type I RH strain or type III CTG strain for 24 h. Cells were syringe lysed and cellular debris pelleted by centrifugation at 1,000 × *g*. The resulting supernatant was centrifuged at 10,000 × *g* to pellet the heavy membrane fraction containing mitochondria and the PVM. Light membranes were pelleted at 100,000 × *g*. (**B**) IFN-γ-treated HeLa cells were infected with type I RH strain or type III CTG strain parasites. The cells were syringe lysed 24 hpi followed by differential centrifugation and immunoblotting for mitochondria (anti-Sam50), the PVM (anti-GRA7) and the parasite membrane (anti-SAG1). LI-COR IRDye 800CW (green) goat anti-rabbit (Sam50 and GRA7 blot) or goat anti-mouse (SAG1 blot) IgG were used as secondary antibodies. (**C**) Model showing affinity purification of ubiquitinated proteins using UBIQAPTURE-Q matrix. IFN-γ-treated HeLa cells were infected with type I RH strain or type III CTG strain parasites. The 10,000 × *g* pellet was lysed and incubated with UBIQAPTURE-Q matrix overnight at 4°C to affinity purify ubiquitinated parasite and host proteins. Affinity purified ubiquitinated proteins were detected by immunoblotting with mouse monoclonal ubiquitin (BML-PW8810-0100; Enzo Life Sciences) antibody. LI-COR IRDye 800CW (green) goat anti-mouse IgG was used as secondary antibody. (**D**) Heat map showing weighted spectra of top *T. gondii* proteins (fold change ≥ 1.5 in CTG versus RH infection) captured by ubiquitin binding matrix. Proteins are ranked by fold difference. Corresponding average fold change. Data are combined from two independent experiments. See also Tables S7 and S8.

Previous studies indicate that ubiquitination of unknown targets on the PVM is the initial step in ATG-mediated growth restriction (39) and ubiquitination is required for subsequent recruitment of LC3 to the PV surrounding *T. gondii* in HeLa cells (58). To identify potential substrates of ubiquitination, we infected IFN-γ activated HeLa cells with type I RH or type III CTG parasites and fractionated cells to enrich the PVM (Fig. 5A). We then subjected these fractions to Western blot analysis using antibodies to the parasite surface protein SAG1, PVM marker GRA7, and host cell mitochondrial protein Sam50. As expected, Sam50 was found in the 1,000 × *g* pellet and the heavy membrane fraction that pelleted at 10,000 × *g* (Fig. 5B). SAG1 was abundant in the 1,000 × *g* pellet where the majority of intact parasites are found, and although it was also detected in the 10,000 × *g* pellet, its abundance was reduced by ~5-fold. Finally, GRA7 was found as an abundant protein in all three fractions, consistent with the partitioning of the PVM into both the 10,000 × *g* and 100,000 × *g* pellets. Western blot analysis of these fractions with the antibody FK2, which recognizes mono- and poly- ubiquitinated proteins, indicated a low abundance of target proteins in the 100,000 × *g* pellet. As such, we focused on the 10,000 × *g* pellet that contains mitochondria as well as components of the PVM, such as MAF1, which mediates association with mitochondria (20).

We utilized a ubiquitin affinity capture resin that binds to mono and poly-ubiquitinated proteins to enrich for ubiquitinated proteins and their interactors. Enriched fractions from two independent replicates were subjected to quantitative mass spectrometry analysis (Fig. 5C). The overall intensity of staining with FK2 was similar in both the input lysate from the 10,000 × *g* pellet as well as the proteins captured on the resin, suggesting a similar overall extent of ubiquitination in cells infected with RH versus CTG strain parasites (Fig. 5C). However, following mass spectrometry and quantitative analysis of the captured proteins, a large number of proteins were differentially enriched from CTG versus RH infected cells (Fig. 5D Data set 2). Many of these proteins are known secretory proteins including those found within the PV (i.e., SRS proteins, BPK, GRAs), on the PVM (i.e., GRAs, ROPs), or exported beyond the vacuole into the host cell (i.e., MAG1) (Data set 2). These proteins were specifically enriched in ubiquitin capture from infected cells but not samples of extracellular parasites processed in parallel (Data set 3). Prominent among the strain-dependent ubiquitin-affinity captured targets are MAG1 and PSD1, which lie within the QTL on chromosome VIII. We also detected MAF1, localized on chromosome II, although it was not enriched in CTG versus RH (Data set 2).

### Testing candidates that mediate susceptibility to LC3 recruitment

**Fig 6:**
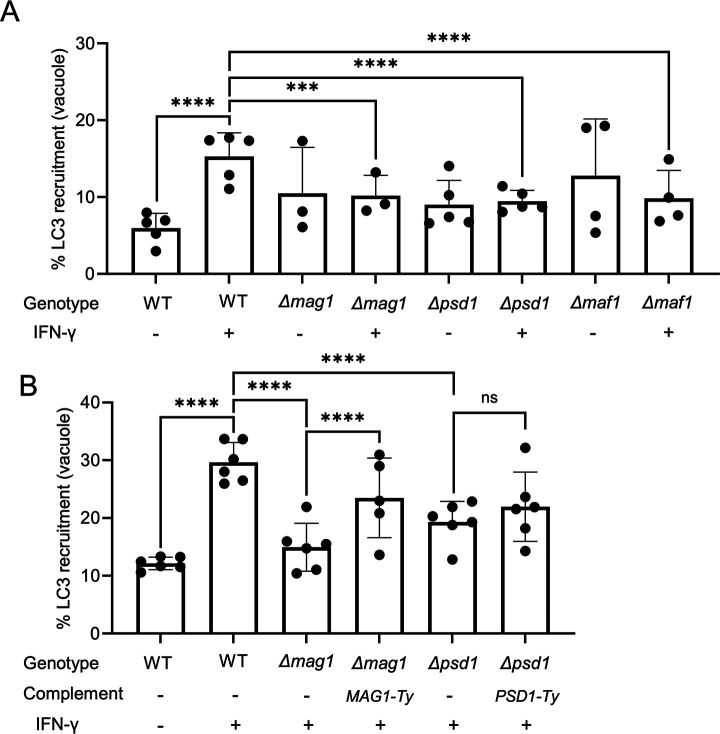
Genetic assessment of candidate proteins that mediate LC3 recruitment to the parasitophorous vacuole. (**A and B**) HeLa cells were treated with or without 100 U/mL IFN-γ for 24 h. Cells were infected at an MOI of 4 with indicated parasite lines on a CTG background for 6 h. Cells were fixed in formaldehyde, stained with mouse anti-SAG1 and rabbit anti-LC3 antibodies followed by goat anti-mouse Alexa Fluor 488 antibody, goat anti-rabbit Alexa Fluor 647 antibody and Hoechst 33342. Samples were imaged with a confocal microscope and the percentage of vacuoles with LC3 recruitment was determined in ImageJ. Data in panels A and B represent means of 3–6 biological replicates based on a minimum of 100 vacuoles counted per replicate. Significance was determined using a two-way analysis of variance comparing variance between experimental conditions and replicates. ns. *P* > 0.05. ***P ≤ 0.001, ****P ≤ 0.0001.

The combination of QTL mapping, evidence for enrichment at the PVM, and ubiquitin affinity capture was used to prioritize a list of candidates for functional testing. We initially focused on the QTL on ChrII where MAF1 was the best candidate. We generated a complete deletion of the *MAF1* locus in the CTG strain using CRISPR/Cas9 combined with two guides that flank the entire locus, as determined by the long-read assembly of the RH strain genome recently generated by PacBio sequencing (https://toxodb.org/toxo/app/record/dataset/DS_413cde922d). Complete deletion of the *MAF1* locus was confirmed by diagnostic PCR (Fig. S1). Deletion of the *MAF1* locus resulted in enhanced staining of LC3 in the absence of IFN-γ treatment compared to wild type cells in two of four replicates (Fig. 6A). In contrast, treatment of the CTG ∆*maf1* strain with IFN-γ resulted in significantly lower recruitment of LC3 compared to wild type cells, although again the experimental replicates were variable (Fig. 6A). Next, we focused on the QTL found on ChrVIII that contains the genes encoding MAG1 and PSD1 (Data set 1). CRISPR/Cas9 was used to generate clean knockouts of each gene in the CTG type III background and these lines were confirmed by diagnostic PCR (Fig. S1). Similar to previous studies described above, treatment of wild type CTG with IFN-γ resulted in enhanced LC3 recruitment, as expected (Fig. 6A). Deletion of either MAG1 or PSD1 in the CTG background did not appreciably change the basal level of LC3 recruitment, but in both cases, loss of the protein significantly decreased the enhanced LC3 recruitment that normally occurs following treatment with IFN-γ (Fig. 6A). Collectively, these studies indicate that the dense granule proteins MAG1 and PSD1 contribute to the enhanced susceptibility of CTG to LC3 recruitment and also suggest a minor role for MAF1.

Given the variability in the data for MAF1, and the complexity of the locus that includes multiple copies of two major alleles, we reasoned that it might be difficult to ascertain a role for this locus by trying to complement the deletion. Instead, we focused on the ∆*psd1* and ∆*mag1* mutants and complemented them using slightly different strategies (Fig. S1). A complementation plasmid for MAG1 was generated using the upstream endogenous promoter region followed by the open reading frame and a C-terminal Ty tag and the DHFR 3′ UTR (Fig. S1). Complemented clones *(*∆*mag1, MAG1-Ty*) were isolated after integration into the *UPRT* locus (Fig. S1). In contrast, due to instability of the flanking regions of *PSD1* in *E. coli*, a PSD1 complementation vector was generated using the *DHFR* promoter to drive expression of C-terminally Ty tagged PSD1. Complemented clones (∆*psd1, PSD1-Ty*) were isolated following CRISPR/Cas9 mediated insertion into the *UPRT* locus (Fig. S1). Immunofluorescence assay (IFA) staining confirmed that the proteins were expressed and secreted to the PV where they occupied a peripheral localization (Fig. S1). Complementation of MAG1 restored the enhanced level of LC3 recruitment seen following IFN-γ treatment, confirming that loss of this protein reduces the susceptibility of CTG to recognition (Fig. 6B). Although a similar trend was observed with compensation of PSD1, it did not achieve statistical significance due to higher and more variable staining with LC3 in the absence of IFN-γ treatment (Fig. 6B). Together, these findings suggest that the enhanced susceptibility of CTG to autophagy-mediated growth restriction is in part due to enhanced recognition of the secreted proteins MAF1, PSD1, and MAG1.

## DISCUSSION

Previous studies have shown that strains of *T. gondii* differ in their susceptibility to ATG-mediated growth restriction in human cells activated by IFN-γ (39, 41). Here we took advantage of a previous genetic cross and used linkage mapping to identify loci that mediate differences in susceptibility to ATG-mediated growth restriction between resistant type I and susceptible type III strains. We focused on recruitment of LC3 that is associated with membrane envelopment and stunting of parasite growth and developed an automated, image-based assay to quantitatively monitor recruitment. Unlike previous studies showing that acute virulence in the mouse is driven by a small number of genes that have major effects, we found two moderate QTLs and three minor QTLs that collectively control susceptibility to LC3 recruitment in IFN-γ-activated human cells. We identified several dense granule proteins encoded by genes within the QTL on ChrVIII that contribute to the enhanced susceptibility of CTG to LC3 recruitment. Collectively, these studies reveal that susceptibility to ATG-mediated growth restriction results from a combination of multiple genes, several of which are resident PV proteins that are differentially recognized by the host, resulting in ATG-mediated growth restriction.

QTL analysis identified multiple genetic loci involved in mediating GFP-LC3 recruitment to the PVM surrounding *T. gondii* in IFN-γ-treated HeLa cells. In addition to the two major QTL peaks on ChrII and ChrVIII, respectively, a third minor peak was observed on ChrVIIb when the ChrII peak was fixed as a covariate. A recent improvement in the genome *T. gondii* assembly has physically linked ChrVIIb with ChrVIII in *T. gondii* (59), although for the purposes of our analysis here, we have drawn the chromosome maps to be consistent with historical representations. The minor peak on ChrVIIb (corresponds to the left end of ChrVIII) may indicate the presence of an independent QTL as the LOD scores drop between the main peak on the right end of ChrVIII and the region on ChrVIIb. Additionally, pairwise two-locus scans identified an additive interaction between markers in the main QTL peaks on ChrII and ChrVIII, and also identified an interactive pairing between markers on ChrVIIa and ChrX. Collectively, these findings suggest that at least five genetic loci play a role in susceptibility to recruitment of GFP-LC3. The multigenic nature of this trait may explain why none of the progeny achieves a GFP-LC3 recruitment phenotype as high as the type III parent. The multigenic nature of LC3 recruitment is in marked contrast to previous studies of acute virulence in the mouse where only a few loci dominate in controlling major phenotypic differences (45, 60). Instead, the difference in LC3 recruitment is more akin to differences in *T. gondii* growth rate that were previously mapped to multiple loci (45) or to differences in T-cell responses to infection by different strains (61).

Previous studies on ATG-dependent growth restriction have defined the kinetics of recruitment of host factors to the PVM surrounding *T. gondii* in IFN-γ-treated HeLa cells. Ubiquitination is the first step in this process, followed by recruitment of adaptors (i.e., p62, NDP52), and finally decoration with LC3 resulting in envelopment by multiple membranes that results in impaired parasite growth (39). Here we focused on LC3 recruitment since this step occurs late in the process and is associated with membrane engulfment and growth restriction of susceptible strains (39). Our findings indicate that susceptibility to LC3 recruitment is mediated by secreted parasite proteins that depend on the Golgi protease ASP5, which processes a subset of secreted dense granule effectors (14). We initially considered an active effector model: type I strains secrete an ASP5 dependent protein that blocks LC3 recruitment. However, this model predicts that LC3 recruitment should increase in the type I ∆*asp5* knockout. However, we observed that LC3 recruitment decreased in ∆*asp5* mutants of both type I strains as well as type II strains. This outcome is consistent with an alternative model: LC3 recruitment depends on differential recognition, with type II and III strains being better recognized, while type I strain is cloaked. In contrast to ASP5, differences in LC3 recruitment did not require the function of MYR1, which is involved in translocation of effectors across the PVM (15, 16). Taken together, these findings suggest that susceptibility to LC3 recruitment is due to differences in recognition of parasite proteins that are resident in the PVM. Our studies implicate MAF1, MAG1 and PSD1 as components that influence susceptibility to LC3 recruitment, although they do not rule out participation of other factors.

To identify potential targets of the initial step of ubiquitination by the host, we employed a sensitive and unbiased strategy to capture proteins based on their interaction with a ubiquitin binding matrix. Ubiquitin affinity capture identified a number of secreted proteins at the PV interface, and many of these showed enhanced capture in CTG-infected cells. Interestingly, the pattern of ubiquitin capture was selective, and some PVM resident proteins (i.e., GRA17, GRA23, MAF1, GRA6, ROP2, ROP4, and ROP7) were abundantly enriched, while others (GRA15, ROP17, and GRA16) were not detected, despite evidence that they can be labeled on the surface of the PVM (15). Among the proteins that were differentially enriched in the pulldowns from CTG infected versus RH infected were MAG1 and PSD1, which are encoded by genes within the QTL on chromosome VIII. We also detected MAF1 in the pulldowns, although it was not differentially captured in a strain-dependent manner. Additionally, we detected a number of lumenal PV proteins (e.g., GRA1, BPK1, and GRA12) and SRS proteins on the surface of the parasite, indicating that the vacuole becomes permeable at some stage. The parasite proteins detected in infected cells are likely the targets of host ubiquitination as only minimal levels of secretory proteins were observed using ubiquitin capture on extracellular parasites, consistent with previous reports (62). An important limitation of our study is that the ubiquitin capture does not reveal the direct targets of ubiquitination, and proteins captured by the matrix might either be authentic targets or proteins that interact with such targets. Our studies also do not define the molecular basis for the strain differences in ubiquitin capture that could arise from differential recognition of different protein alleles by the host ubiquitination pathway, differences in expression level, or interactions with authentically ubiquitinated targets. Future studies to reconstitute expression of different alleles of susceptibility determinants by complementation in the endogenous locus could provide better insight into how expression levels versus allelic differences contribute to susceptibility. Regardless of the precise mechanism for enrichment in ubiquitin capture, these studies suggest that differential recognition of parasite proteins at the PVM gives rise to ATG-mediated growth restriction.

The profile of proteins that are ASP5 dependent and MYR1 independent fit MAF1 that encodes multiple copies of the paralog MAF1b, which is responsible for mitochondrial recruitment to the PVM, and MAF1a, the function of which is unknown (19, 20). Deletion of the entire *MAF1* locus in the type III CTG strain increased susceptibility to LC3 recruitment, which is consistent with the ChrII QTL mapping, where the CTG allele is protective. In contrast, two other genes of ChrVIII that encode proteins that are localized to the PV were implicated in the enhanced susceptibility of CTG to ubiquitination and LC3 recruitment. MAG1 has previously been shown to reside in the PV (56) and to interact with resident PVM proteins (53). It has also recently been shown to cross the PVM and accumulate in the cytosol of the host cell where it inhibits IL-1β production (56). Export of MAG1 is not MYR1 dependent (56), and it contains several TEXEL motifs consistent with being detected in the ASP5 proteome (12). Deletion of MAG1 in the type III CTG strain significantly reduced LC3 recruitment, and this defect was complemented by restoring expression of MAG1, indicating that it participates in the differential recognition by the host. Separately, PSD1 was also shown to mediate susceptibility to ubiquitination and LC3 recruitment, and in its absence, recruitment was significantly decreased. Complementation failed to restore this defect, which could be due to variability in the assay or inefficient expression due to less efficient export from expression from the *UPRT* locus and under a heterologous promoter. PSD1 is a dense granule protein that is secreted into the PV, where it associates with the PVM (57). PSD1 has a predicted internal TM domain, suggesting it may be a resident PVM protein that is partially exposed to the host cytosol and thus potentially recognized by the host. Consistent with this, PSD1 was also detected in permissive biotin ligase experiments using resident PVM proteins (53). The basis for why type II and III strains are readily recognized, while type I strains are effectively cloaked, remains uncertain. All three proteins studied here contain low levels of polymorphism, although the allelic patterns do not suggest a shared basis for susceptibility (i.e., susceptible type II and III strains do not share a common type). Alternatively, strain differences in recognition may relate to differences in expression level or surface exposure at the PVM.

Collectively, our studies reveal that susceptibility to ATG-dependent growth restriction is a multigenic process that depends on at least five loci in the parasite. Unlike previous effectors that have been shown to antagonize host functions (8), susceptibility to LC3 recruitment and subsequent ATG-dependent growth restriction results from differential recognition of susceptible strains rather than active inhibition by resistant ones. Finally, our studies show that differential susceptibility is partially dependent on several dense granule proteins that are secreted into the PV and which may transit through or become resident in the PVM, thus being exposed to recognition by the host. These findings provide new insight into cell-autonomous immunity by demonstrating that susceptibility to recruitment of ATG mediators such as LC3 is mediated by multiple loci in *T. gondii*.

## MATERIALS AND METHODS

### Parasite strains and cell culture

The parental *T. gondii* strains used in the type I (GT1-FUDR) × type III (CTG-ARA) genetic crosses, along with 34 unique F1 progeny, were previously described (63). To examine the role of MYR1 in GFP-LC3 recruitment, we used the parental strain RH∆*hxgprt* and the mutant RH*∆myr1* that was derived in this background (16), as well as the parental type II strain ME49-FUDR (64) and a corresponding mutant ME49*∆myr1* made in a similar background (16). To examine the role of ASP5 in GFP-LC3 recruitment, we compared the parental strains RH∆*ku80* (65) and ME49-FUDR (66) to the mutants RH∆*ku80*∆*asp5* (12) and ME49∆*asp5* (10). The ME49-Fluc strain expressing firefly luciferase was described previously (67), and additional wild-type strains for ME49 and CTG were obtained from the American Type Culture Collection. All strains used in the present study are listed in Table S6. Strains of *T. gondii* were serially passaged in human foreskin fibroblasts maintained in Dulbecco's minimal essential medium (DMEM) (glutamine [10 mM] and gentamycin [10 µg/mL]) supplemented with 10% fetal bovine serum (FBS, Gibco). Infected monolayers were scraped, syringe passed through a 23-g needle, and host cell debris removed by filtration through a 3.0-µm polycarbonate filter prior to use for each assay. HeLa cell lines expressing GFP fused to LC3 were cultured in minimal essential medium (MEM) supplemented with 10% FBS, 4 mM L-glutamine, and 10 mM HEPES solution. GFP-LC3 plasmid was maintained using G418 (50 µg/mL) in the culture media. All parasite and host cell cultures were maintained in a 37°C incubator with 5% CO_2_ and were confirmed to be mycoplasma free using the e-Myco Plus kit (Intron Biotechnology).

### Generation of knockouts in the CTG strain

Plasmids were assembled from DNA fragments by the Gibson method (68). All primers were synthesized by Integrated DNA Technologies. CRISPR/Cas9 plasmids used in this study were derived from the single-guide RNA (sgRNA) plasmid pSAG1:CAS9-GFP, U6:sgUPRT by Q5 site-directed mutagenesis (New England BioLabs) to alter the 20-nt sgRNA sequence, as described previously (69). Primers used in this study are listed in Table S7, and plasmids are listed in Table S8. Gene disruptants and complemented lines in *T. gondii* were generated using CRISPR/Cas9, as described previously (69). Genomic sequences from ToxoDB.org were used to design flanking regions for generating knockout plasmids to replace the coding region with the pyrimethamine resistant DHFR allele (Fig. S1). We generated several transgenic knockout lines in the type III CTG strain including the mutants CTG ∆*mag1*, CTG ∆*psd1*, and a complete *MAF1* locus deletion named CTG∆*maf1*, as described in Fig. S1.

### Complement generation

#### *MAG1* complementation

Freshly harvested CTG strain parasites were resuspended in PBS containing 1× Taq standard reaction buffer (NEB) and 250-µg/mL proteinase K and incubated at 37°C for 1 h, 56°C for 1 h, and 95°C for 10 min. The *MAG1* sequence was amplified from the isolated genomic DNA to generate a fragment that included the open reading frame and 1,404 bp of upstream sequence using primers listed in Table S7 and PrimeSTAR GXL DNA Polymerase. The entire *MAG1* gene with upstream promoter was cloned into a pUPRT targeting plasmid upstream of an in-frame 2×Ty1 tag, a 781-bp sequence corresponding to the 3′ untranslated region of DHFR, and a chloramphenicol acetyltransferase gene using primers listed in Table S7 using a Gibson Assembly kit (NEB). The complementation vector was electroporated into CTG Δ*mag1* parasites together with a CRISPR/Cas9 sgRNA to the *UPRT* locus (Addgene 54467) (70) followed by selection with 20 µM chloramphenicol and 10 µM fluorodeoxyuracil. Complemented clones *(*∆*mag1* and *MAG1-Ty*) were generated via expansion after limiting dilution.

#### PSD1 complementation

RNA was isolated from CTG tachyzoites using an RNeasy mini kit (Qiagen), and cDNA was generated using an iScript cDNA Synthesis Kit according to the manufacturer’s protocol. The *PSD1* coding sequence was amplified using primers listed in Table S7 and PrimeSTAR GXL DNA Polymerase (Takara). The DHFR promoter sequence was inserted upstream of the *PSD1* open reading frame that included an in-frame 2×Ty1 tag, followed by a 781-bp sequence corresponding to the 3′ untranslated region of DHFR. The complementation vector was amplified with primers listed in Table S7 from the UPRT homology arms using PrimeSTAR GXL DNA Polymerase. The linearized complement vector and CRISPR/Cas9 sgRNA UPRT plasmid (Addgene 54467) (70) were electroporated into CTG Δ*psd1* parasites. Insertion into the *UPRT* was selected for with 20 µM chloramphenicol and 10 µM fluorodeoxyuracil. Complemented clones *(*∆*psd1* and *PSD1-Ty*) were generated via expansion after limiting dilution.

### Parasite transfection

Following natural egress, freshly harvested parasites were transfected with plasmids using protocols previously described (69). In brief, ~2 × 10^7^ extracellular parasites were resuspended in 370 µL cytomix buffer, were mixed with ~30 µL purified plasmid or amplicon DNA in a 4-mm gap BTX cuvette and electroporated using a BTX ECM 830 electroporator (Harvard Apparatus) using the following parameters: 1,700 V, 176-μs pulse length, two pulses, and 100-ms interval between pulses. Transgenic parasites were isolated by outgrowth under selection with mycophenolic acid (25 µg/mL) and xanthine (50 µg/mL), pyrimethamine (3 mM), chloramphenicol (20 µM), and 5-fluorodeoxyuracil (10 µM) (Sigma), as needed. Stable clones were isolated by limiting dilution on HFF monolayers grown in 96-well plates.

### Image-based GFP-LC3 recruitment assay

We used a previously described GFP-LC3 recruitment assay (43) with the following modifications: assays were completed in 96-well μClear assay plates (Greiner Bio-One, #655090). After culture for 24 h, GFP-LC3 HeLa cells were activated for 24 h with human interferon gamma (IFN-γ; 100 U/mL, 100 µL volume; R&D Systems) or left untreated. Following activation, 5 × 10^4^ *T. gondii* tachyzoites were added to each well (100 µL volume, 200-µL total well volume) and allowed to invade for 2 h. At 2 h post-invasion, plates were rinsed three times in DMEM; fresh media were added to all wells (200-µL volume); and plates were returned to 37°C for 4 h prior to fixation for IFA. Cells were fixed in 4% formaldehyde, permeabilized with 0.05% saponin, and blocked with 2.5% FBS/2.5% goat serum prior to staining for immunofluorescence. *T. gondii* containing vacuoles were stained with the anti-RH antibody (polyclonal rabbit serum) that cross reacts with all strains of *T. gondii* and visualized with Alexa Fluor 594 (Invitrogen). GFP-LC3 was localized using a mouse monoclonal antibody 3E6 (#A11120, Life Technologies) followed by detection with Alexa Fluor 488 (Invitrogen). Imaging was completed on a Cytation 3 Multimode plate reader (BioTek). Positive recruitment events were defined by signal overlap of GFP-LC3-positive cells (green cells >55,000 units) and parasite-positive objects (red >5,000 relative fluorescence units, parasite size range 5–11 µm). Parasites (red >5,000 relative fluorescence units) that did not overlap with mean background levels of GFP-LC3 signal (green <5,000 relative fluorescence units) were considered non-invaded and therefore not counted.

### Immunofluorescence assay for LC3 recruitment in type III parasites

HeLa cells were stimulated with IFN-γ (100 U/mL) for 24 h, infected with tachyzoites of the type III *T. gondii* strain CTG, and washed 2 h post-infection to remove extracellular parasites. Control cells were grown in the absence of IFN-γ and infected in a similar manner. The cells were fixed in 4% formaldehyde 6 h postinfection, permeabilized with 0.02% saponin, and stained sequentially with primary and secondary antibodies. Samples were subsequently incubated in wash buffer containing 1:2,000 rabbit anti-LC3 antibody (PM036, MBL International Corporation) and 1:1,000 mouse anti-SAG1 antibody (DG52) for 1 h. Samples were washed four times in wash buffer for 5 min each wash and incubated in wash buffer containing 1-µg/mL Hoechst 33342, 1:2,000 goat anti-rabbit Alexa Fluor 647 antibody and 1:2,000 goat anti-mouse Alexa Fluor 488 antibody. Samples were washed four times in wash buffer for 5 min each wash and mounted on slides with Prolong Gold Antifade Reagent. Samples were imaged through a Plan-Neofluar 20× (Zeiss) objective on a Zeiss Observer Z1 inverted microscope (Carl Zeiss Inc) equipped with a Colibri 7 (Zeiss) light source and ORCA-ER digital camera (Hamamatsu Photonics). Images were acquired in ZEN Blue (version 2.5) software. Images were blinded, and LC3 staining of vacuoles was quantified manually using ImageJ. The percentage of positive parasites was determined from 300 or more PV on three separate coverslips per group.

### QTL mapping

QTL analysis of the recruitment of GFP-LC3 to the *T. gondii* PVM was conducted using J/qtl (version 1.3.5) software (71) to correlate phenotype with genotype for each individual progeny of the *T. gondii* type I (GT1-FUDR) × type III (CTG-ARA) genetic cross (63). We used the original assembly of the *T. gondii* genome consisting of 14 chromosomes (22), although subsequent studies have shown that chromosome VIIb is actually fused to chromosome VIII (72). Here it is referred to as ChrVIIb with the acknowledgment that it actually represents the left end of ChrVIII. QTLs associated with ATG effector recruitment were identified using a single-genome scan approach with each QTL needing a minimum threshold LOD score of >3 to define major peaks. Confirmation of QTL peaks was based on 1,000 random permutations generating a minimum likelihood score of (*P* = 0.05). Covariate analysis using single-genome scans were completed to evaluate the interaction and influence of the two QTLs to the ATG recruitment and to ensure identification of any minor QTLs. All covariate analyses and QTLs identified were subject to minimum thresholds described above. Pairwise interactions were evaluated using two-genome scan analysis in J/qtl including additive versus full and interactive versus full. Two-genome scans are based on the same statistical cutoffs identified by the single-genome scan described above.

### Ubiquitin capture

HeLa cells were stimulated with IFN-γ (100 U/mL) for 24 h and infected with wild-type III CTG strain versus type I RH strain parasites. Cells were washed 2 h post-infection to remove extracellular parasites and scraped 24 h post-infection in IC buffer (KCl 142 mM, NaCl 5 mM, MgCl_2_1 mM, D-glucose 5.6 mM, EGTA 2 mM, HEPES 25 mM, pH 7.4) followed by syringe lysing by sequentially passing through 20-g, 23-g, and 25-g needles. The cells were sequentially pelleted at 1,000 × *g* (5 min), 10,000 × *g* (30 min), and 100,000 × *g* (1 h), and extracts were used for Western blot analysis. Proteins were extracted from the pellets in 0.5% NP-40 lysis buffer (NaCl 150 mM, Tris-Cl 50 mM, protease inhibitor cocktail [Roche], N-ethylmaleimide 10 mM, EDTA 1 mM). In parallel, a comparable number of freshly harvested parasites were processed in the absence of host cells in order to monitor ubiquitination of proteins that are intrinsic to the parasite. The extracted proteins were incubated with UBIQAPTURE-Q matrix (BML-UW8995A-0001, Enzo Life Sciences) at 4°C overnight for affinity capture of ubiquitinated proteins. The UBIQAPTURE resins bind to mono- and poly-ubiquitinated proteins of different lysine linkages. The matrix was washed four times with PBS to remove non-specific proteins and was stored at −80°C until analysis. Experiments were repeated two independent times and processed in parallel.

### Western blotting

Samples were boiled in non-reducing SDS sample buffer and resolved on 12% acrylamide gels. To examine cell fractions, resolved proteins were transferred on to the nitrocellulose membranes and probed with rabbit polyclonal GRA7 (73), mouse monoclonal SAG1 (provided by John Boothroyd, Stanford University), rabbit monoclonal Sam50 (ab249440, Abcam), or mouse monoclonal ubiquitin (BML-PW8810-0100, Enzo Life Sciences) antibody followed by incubation with IRDye conjugated secondary antibodies (LI-COR).

### Mass spectrometry identification of affinity captured proteins

Samples were submitted to the Proteomics and Metabolomics Facility, Center for Biotechnology, University of Nebraska-Lincoln for mass spectrometry (MS) analysis. In brief, samples were eluted from the beads in NuPAGE LDS sample buffer containing 10 mM dithiothreitol (DTT), heated to 95°C and resolved on Bolt 12% Bis-Tris-Plus gels (Thermo Fisher Scientific) in MES SDS running buffer. Samples were electrophoresed into the top of the gel, fixed in methanol:acetic:water (40:10:50), stained with Coomassie blue 250, then destained before excising the stained band for further processing. Gel bands were reduced with DTT in ammonium bicarbonate, pH 8, for 1.5 h at 37°C, acidified to pH 6, and alkylated with N-ethylmaleimide. Gel slices were digested with trypsin, peptides extracted, dried down, and redissolved in 5% acetonitrile and 0.2% formic acid. Samples were resolved by nanoLC-MS/MS using a 2-h gradient on a 0.075 mm × 250 mm CSH C18 column (Waters Corp, Milford, MA) connected to an Orbitrap Eclipse mass spectrometer. MS/MS samples were analyzed using Mascot (version 2.6.2; Matrix Science, London, UK). Mascot was set up to search the cRAP_20150130.fasta (124 entries); Custom_20210401.fasta; ToxoDB-28_TgondiiME49_AnnotatedProteins_20160816.fasta; uniprot-human 20201207 database assuming the digestion enzyme trypsin. Mascot was searched with a fragment ion mass tolerance of 0.060 Da and a parent ion tolerance of 10.0 parts per million (PPM). Deamidation of asparagine and glutamine, oxidation of methionine, GG of lysine, phosphorylation of serine, threonine, and tyrosine, *N*-ethyl-maleimide of cysteine, and *N*-ethylmaleimide + water of cysteine and lysine were specified in Mascot as variable modifications. Scaffold (version Scaffold_4.8.9; Proteome Software Inc., Portland, OR) was used to validate MS/MS-based peptide and protein identifications. The ubiquitinated parasite and host proteins identified by MS were analyzed using Scaffold (version 4.0, using the following criteria: number of peptides 2, protein threshold 99%, and peptide threshold 95%) (74). We analyzed the two replicate experiments in combination to identify parasite proteins that were on average more than 1.5-fold abundant (based on normalized weighted spectra) in CTG strain versus RH strain infection. Heat maps showing the spectral abundance of the shortlisted proteins in each strain and the fold spectral abundance in CTG versus RH infection were created in GraphPad Prism (version 8).

### Statistical analysis

Statistical analysis was performed using Prism (version 8, GraphPad Software Inc.), and details of the respective test and significance values are described in the figure legends.

## SUPPLEMENTAL MATERIAL

Data Set S1 mbio.02595-23-s0001.xlsx Candidate genes shared between the two major QTLs and known mediators of export of PVM localized proteins.

Data Set S2 mbio.02595-23-s0002.xlsx Ubiquitinated proteins from intracellular parasites identified by affinity capture.

Data Set S3 mbio.02595-23-s0003.xls Ubiquitinated proteins from extracellular parasites identified by affinity capture.

Fig. S1 mbio.02595-23-s0004.pdf Strategy for generation of knockout and complemented lines of CTG strain.

Supplemental Tables mbio.02595-23-s0005.pdf Tables S1-S8.

ASM does not own the copyrights to Supplemental Material that may be linked to, or accessed through, an article. The authors have granted ASM a non-exclusive, world-wide license to publish the Supplemental Material files. Please contact the corresponding author directly for reuse.
