# Supplementary material for: Susceptibility of Toxoplasma gondii to autophagy in human cells relies on multiple interacting parasite loci
Source: mBio. 2023 Dec 14;15(1):e02595-23. doi: 10.1128/mbio.02595-23 (PMC10790690; doi:10.1128/mbio.02595-23)
Supplement: Fig. S1 — Strategy for generation of knockout and complemented lines of CTG strain. [file mbio.02595-23-s0004.pdf]

Supplemental Materials

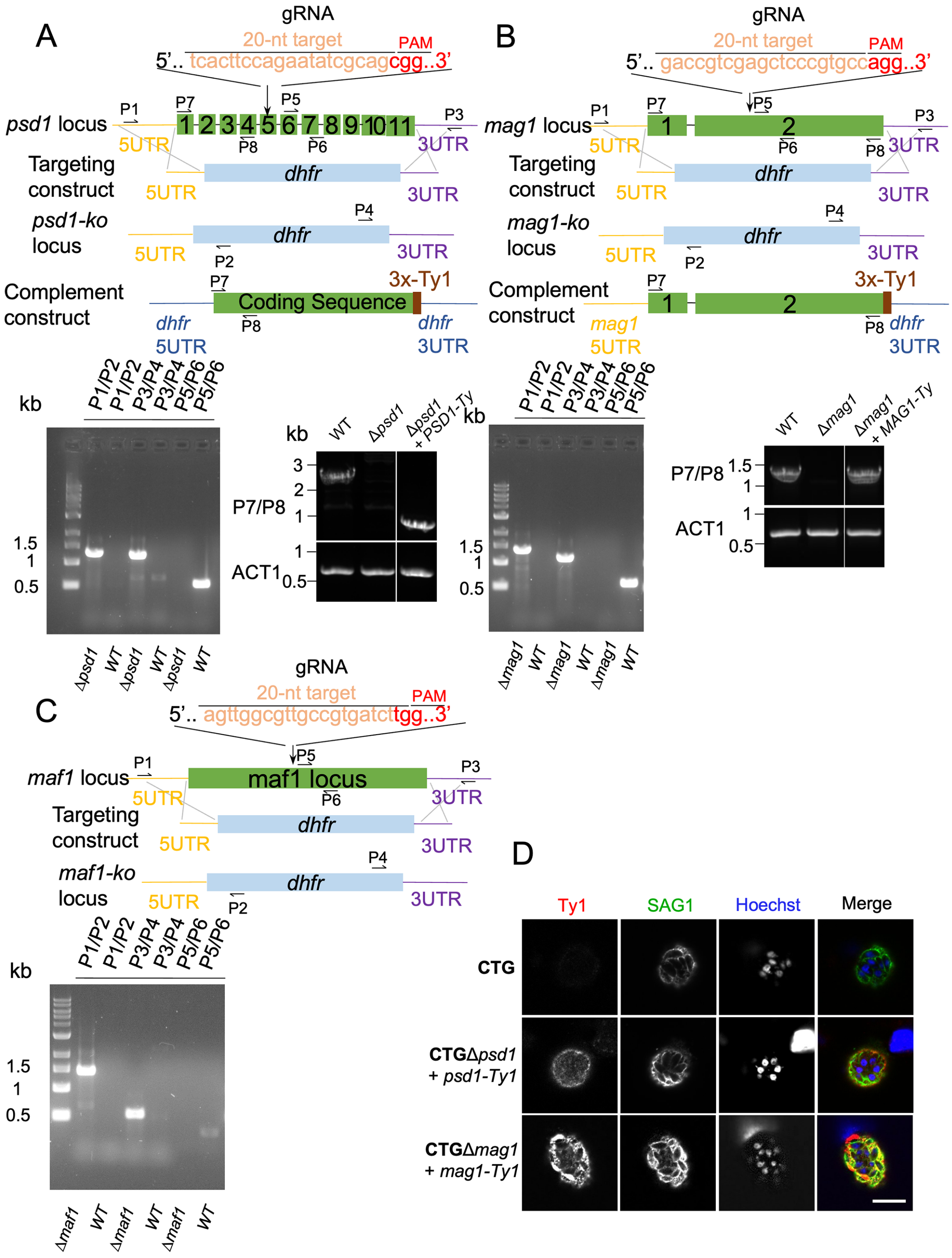

Figure S1 Strategy for generation of knockout and complemented lines of CTG strain.

(A-C) Schematic representation of the strategy for CRISPR/Cas9-mediated gene deletion used to generate transgenic strains used in this study. A single sgRNA expressing CRISPR/Cas9 (gRNA) plasmids targeting the middle of the genes were used to mediate a knockout by homologous

recombination. Targeting constructs consisted of a selection cassette (Δpsd1-dhfr, Δmag1-dhfr, Δmaf1-dhfr) and long homology flanks (~500bp) immediately upstream of the translation initiation site (left arm) and downstream of the stop codon (right arm) as homologous arms to the flanking regions of the GOI. Relying on the novel RH-88 Genome Sequence assembly conducted using PacBio (GCA_013099955.1) 5 UTR of the first MAF1 gene within the locus and 3UTR of TGGT1_ 221160 genes were used as homology flanks (~500bp) for the entire MAF1 locus. (A-B) Following knockout generation, knockouts were complemented with a Ty1 tagged coding sequence driven by a DHFR promoter (A) or a Ty1 tagged genomic sequence driven by the endogenous promoter. Genomic insertion was driven by homologous recombination into the *UPRT* locus by contransfection with a UPRT sgRNA plasmid CRISPR/Cas9 plasmid (Addgene 5446). Diagnostic PCR results (bottom left panel) to verify locus disruption. The priming sites for PCR primers are indicated: P1/P2 and P3/4 confirm integration of left and right homologous arms, respectively; P5/6 examines the integrity of the endogenous gene. A successful knockout clone gave positive PCR products in P1/P2 and P3/4 but no product in P5/6, whereas the wild type parasites gave the opposite. Diagnostic PCR results (bottom right panel) to verify complementation. The priming sites for PCR primers are indicated: P7/P8 confirm integration. Both wild type and a successful complement clone gave positive PCR products in P7/8 whereas the knockout parasites did not. ACT1 amplification serves as an amplification positive control. See also Table S7 for oligonucleotide sequences. (D) Localization of Ty1 tagged proteins in complemented knockouts. HeLa cells were infected at an MOI of 0.5 with indicated parasite lines for 48h. Cells were fixed in formaldehyde, stained with mouse anti-Ty1 and rabbit anti-SAG1 antibodies followed by goat anti-mouse Alexa Fluor 568 antibody, goat anti-rabbit Alexa Fluor 488 antibody and Hoechst 33342. Samples were imaged with a confocal microscope. Scale bar: 10 μm.
