# Supplementary material for: Susceptibility of Toxoplasma gondii to autophagy in human cells relies on multiple interacting parasite loci
Source: mBio. 2023 Dec 14;15(1):e02595-23. doi: 10.1128/mbio.02595-23 (PMC10790690; doi:10.1128/mbio.02595-23)
Supplement: Supplemental Tables — Tables S1-S8. [file mbio.02595-23-s0005.pdf]

Table S1 GFP-LC3 recruitment in naïve and hIFN-γ-activated HeLa cells for type I x type III genetic cross

	Naïve			hIFN-γ (100 U/mL)		
**Strain**	**GFP-LC3 %+**	**SEM**	**N**	**GFP-LC3 %+**	**SEM**	**N**
CTG-ARA	2.16	0.26	38	29.47	1.18	38
GT1-FUDR	0.68	0.12	16	2.19	0.29	16
A9SF	2.70	0.43	10	12.88	1.55	10
G2AF	2.93	0.41	6	9.85	1.05	6
G2SF	0.75	0.10	6	1.80	0.31	6
C285-1	3.53	0.57	6	8.52	0.80	6
C285-4	3.40	0.58	6	8.27	0.53	6
C285-11	4.57	0.52	6	10.17	1.27	6
A6AF	3.72	0.65	6	7.23	1.21	6
C11AF	5.28	0.45	6	9.82	1.10	6
E7SF	3.63	0.50	6	7.45	0.92	6
D9AF	3.42	0.59	10	10.95	1.56	10
B4SF	2.80	0.57	8	11.20	1.52	8
H7AF	4.33	0.44	6	8.87	1.00	6
E8SF	3.48	0.61	10	11.85	1.43	10
B10AF	1.27	0.31	6	9.07	0.98	6
C285-10	0.90	0.20	6	2.12	0.43	6
C295-31	0.95	0.12	8	7.66	1.11	8
C295-3	0.55	0.11	6	3.45	0.30	6
C7SF	1.28	0.32	6	3.08	0.40	6
C295-5	1.48	0.47	6	3.45	0.67	6
C295-27	2.08	0.49	6	3.55	0.46	6
C295-29	1.58	0.62	6	3.13	0.96	6
E6AF	2.25	0.25	6	3.78	0.65	6
C285-13	1.13	0.34	6	3.27	0.41	6
C295-2	1.53	0.18	10	6.82	1.00	10
C285-31	0.52	0.09	6	4.58	1.32	6
C285-38	1.28	0.16	6	5.93	1.42	6
C285-42	0.88	0.19	6	2.73	0.53	6
C285-50	0.77	0.12	12	6.73	1.14	12
C285-51	0.97	0.12	6	5.38	1.29	6
C285-62	1.10	0.21	6	9.55	1.81	6
M12AF	0.77	0.17	12	6.39	1.25	12
A9AF	0.50	0.20	6	2.70	0.38	6
C295-P1A5	0.28	0.21	6	2.23	0.53	6
C295-P1D7	0.67	0.10	6	4.27	0.30	6

Table S2 GFP-LC3 recruitment in naïve and hIFN-γ-activated HeLa cells to evaluate ASP5 role in ATG effector recruitment

	Naïve			hIFN-*γ* (100 U/mL)		
**Strain**	**GFP-LC3 % +**	**SEM**	**N**	**GFP-LC3 % +**	**SEM**	**N**
RH*Δku80*	1.77	0.17	6	7.28	0.65	6
RH*Δku80Δasp5*	1.81	0.22	15	2.86	0.47	15
ME49	0.90	0.38	3	13.67	1.45	3
ME49*Δasp5*	1.04	0.49	3	3.73	2.24	3

Table S3 GFP-LC3 recruitment in naïve and hIFN-γ-activated HeLa cells to assess MYR1 role in ATG effector recruitment.

	Naïve			hIFN-*γ* (100 U/mL)		
**Strain**	**GFP-LC3 %+**	**SEM**	**N**	**GFP-LC3 %+**	**SEM**	**N**
RHmCherry	1.783	0.28	6	7.47	0.61	6
RH*Δmyr1*	2.367	0.51	6	5.87	0.73	6
RH*Δmyr1*::MYR1	1.367	0.24	6	5.67	0.95	6
ME49-FUDR	3.567	0.41	6	21.63	1.80	6
ME49-Fluc	2.272	0.43	9	11.57	1.22	9
ME49*Δmyr1*	2.088	0.44	9	19.44	0.90	9

Table S4 Summary of single genome scan effects for ATG recruitment susceptibility in *T. gondii*.

Marker	Effect	Chr[a]	Position	LODb	Significance
W35487	Main	II	4.0 cM	3.325	*P* = 0.017
AK113	Main	VIII	11.0 cM	3.974	*P* = 0.002
L363	Covariate (ChrII)	VIIb	2.0 cM	3.117	*P* = 0.080
AK50	Covariate (ChrII)	VIII	12.0 cM	6.818	*P* = 0.000
W35487	Covariate (ChrVIII)	ChII	4.0 cM	6.169	*P* = 0.002

[a] Chr, chromosome
[b]LOD, logarithm of odds

Table S5 Summary of two locus genome scan effects for ATG recruitment susceptibility in *T. gondii*

Marker Pair	Effect	Chr[a]	Position	LOD[b]	Significance
KT-L379A/AK113	Full	II/VIII	3.0 cM/11.0 cM	10.144	*P* = 0.000
L31-T3/AK113	Additive	II/VIII	5.0 cM/11.0 cM	9.676	*P* = 0.000
SAG4/L366	Interactive	VIIa/X	1.0 cM/9.0 cM	4.032	*P* = 0.344
SAG4/AK132	Interactive	VIIa/X	1.0 cM/7.0 cM	1.33	*P* = 0.988

[a] Chr, chromosome
[b]LOD, logarithm of odds

Table S6 *Toxoplasma gondii* lines used in this study.

Line	Genotype	Source
GT1-FUDR	GT1 FUDR[r] chemical mutagenesis	(1)
RHΔku80	RHΔ*hxgprtΔku80*	(2)
RHΔku80Δasp5	RHΔ*hxgprtΔku80Δasp5*	(3)
RHΔhxgprt	RHΔ*hxgprt*	(4)
RHΔmyr1	RHΔ*hxgprt*Δmyr1	(4)
ME49	Wild type II	ATCC 50611
ME49-FUDR	ME49 FUDR[r] chemical mutagenesis	(5)
ME49Δmyr1	ME49 *Δhxpgrt::FLUC, Δmyr1*	(4)
ME49-Fluc	ME49 *Δhxpgrt::FLUC*	(6)
ME49Δasp5	ME49Δasp5	(7)
CTG	Wild type III	ATCC 50842
CTG-ARA	CTG AraA[r] chemical mutagenesis	(1)
CTG Δmaf1	CTG *Δmaf1::DHFR-TS*	This study
CTG Δmag1	CTG *Δmag1::DHFR-TS*	This study
CTG Δpsd1	CTG *Δpsd1::DHFR-TS*	This study
CTG Δmag1, MAG1-Ty	CTG *Δmag1::DHFR-TS, MAG1-Ty2-DHFR, Cm*	This study
CTG Δpsd1, PSD1-Ty	CTG *Δpsd1::DHFR-TS, uprt:: DHFR-PSD1-Ty2-DHFR, Cm*	This study

Table S7 Oligonucleotides used in this study.			
OligoNT	Sequence (5' – 3')	Usage	Source
sgRNA_R	aacttgacatccccatttac	Universal reverse primer for mutating sgRNA protospacer sequence on p*SAG1*:*CAS9-GFP*, *U6*:sg*UPRT*	(Shen et al., 2014); IDT
sgMAF1 (3') F	agttggcgttgccgtgatct GTTTTAGAGCTAGAAATAGC	Used with sgRNA_R for generating p*SAG1*:*CAS9-GFP*, *U6*:sgMAF1	This study; IDT
pUC19-MAF1-KO-backbone-F	gacaagacagtgaacacgtgacgttacccaacttaatcgccttg	Primer set used to generate *the backbone from pUC19 for building pMAF1-KO-DFHR-construct by Gibson assembly*	This study; IDT
pUC19-MAF1-KO-backbone-R	gaatgactttctgcgtatgcagtgaaattgttatccgctcac		This study; IDT
MAF1-3UTR-F	CATGGTCATAGCTGTTTCCTGGACTCGTCTCACAGAAAAACACACTGC	Primer set used to generate MAF1 3UTR amplicon from T. gondii RH strain genomic DNA for building pMAF1-KO-DFHR-construct by Gibson assembly	This study; IDT
MAF1-3UTR-R	GTGAGCGGATAACAATTTCACtgcatacgcagaaagtcattc		This study; IDT
MAF1-5UTR-F	CAAGGCGATTAAGTTGGGTAACGTCACGTGTTCACTGTCTTGTC	Primer set used to generate	This study; IDT

MAF1-5UTR-R	CActggccgtcgttttacacatcTGGGAGCACTCCATTTGTCCAC	MAF1 5UTR amplicon from T. gondii RH strain genomic DNA for building pMAF1-KO-DFHR-construct by Gibson assembly	This study; IDT
MAF1-KO-DHFR-F	GTGGACAAATGGAGTGCTCCCAgatgtgtaaaacgacggccagTG	Primer set used to generate of DHFR resistance cassette from pFloxed DHFR-TS* construct for building pMAF1-KO-DFHR-construct by Gibson assembly	This study; IDT
MAF1-KO-DHFR-R	GCAGTGTGTTTTTCTGTGAGACGAGTCCAGGAAACAGCTATGACCATG		This study; IDT
Δ*MAF1* P1_F	taccagtcatggacgagatcg	Primer set used to detect 5' integration of DHFR, into MAF1 locus WT =null; KO = 1400 bp	This study; IDT
Δ*MAF1* P2_R	gcagttcattgtctaggtacgacatttc		This study; IDT
Δ*MAF1* P3_F	gtgcgtgtcattgattcgtgag	Primer set used to detect 3' integration of DHFR, into MAF1 locus WT =null; KO = 550 bp	This study; IDT
Δ*MAF1* P4_R	catggtcatagctgtttcctgg		This study; IDT
Δ*MAF1* P5_F	ccggctgtcctttctgtttg	Primer set used to detect MAF1 WT = 220 bp; KO = null	This study; IDT
Δ*MAF1* P6_R	agatcacggcaacgccaact		This study; IDT
sgPSD1 (3') F	gTCACTTCCAGAATATCGCAG GTTTTAGAGCTAGAAATAG	Used with sgRNA_R for generating p*SAG1*:*CAS9*-	This study; IDT

		GFP, *U6*:sgPSD1	
pUC19-PSD1-KO-backbone-F	CGTTTCTTCCCGTGTAACAGCGTTACCCAACTTAATCGCCTTG	Primer set used to generate *the backbone from pUC19 for the pPSD1-KO-DFHR-construct* for building	This study; IDT
pUC19-PSD1-KO-backbone-R	ATCGCGTTCGAGACTGCTTCGTGAAATTGTTATCCGCTCA		This study; IDT
PSD1-5UTR-F	CAAGGCGATTAAGTTGGGTAACGCTGTTACACGGGAAGAAACG	Primer set used to generate PSD1 5UTR amplicon from T. gondii RH strain genomic DNA	This study; IDT
PSD1-5UTR-R	ActggccgtcgttttacacatGTTGTTTGCAAGACTCGTAC		This study; IDT
PSD1-DHFR-KO-F	GTACGAGTCTTGCAAACAACatgtgtaaaacgacggccagT	Primer set used to generate of DHFR resistance cassette from pFloxed DHFR-TS* construct for the pPSD1-KO-DFHR-construct	This study; IDT
PSD1-DHFR-KO-R	GTGAACTATCGGTGCTCTCTCCAGGAAACAGCTATGACCATG		This study; IDT
PSD1-3UTR-KO-F	CATGGTCATAGCTGTTTCCTGGAGAGAGCACCGATAGTTCAC	Primer set used to generate PSD1 3UTR amplicon from T. gondii RH strain genomic DNA	This study; IDT
PSD1-3UTR-KO-R	TGAGCGGATAACAATTTCACGAAGCAGTCTCGAACGCGAT		This study; IDT
ΔPSD1- P1_F	TGAAACGAGCTGGTTCTCAC	Primer set used to detect 5’ integration of DHFR, into PSD1 locus WT =null; KO = 1300 bp	This study; IDT
ΔPSD1- P2_R	taccagtcatggacgagatcg		This study; IDT
ΔPSD1- P3_F	TTCCTGTTGAGAAAGCGGTTC	Primer set used to detect 3’ integration	This study; IDT

ΔPSD1- P4_R	CGCAGGCGAAATCACGTATATG	of DHFR, into PSD1 locus WT =null; KO = 1200 bp	This study; IDT
ΔPSD1- P5_F	GGTAGCTATGACTGTGAACG	Primer set used to detect PSD1 WT = 450 bp; KO = null	This study; IDT
ΔPSD1- P6_R	CAACAGTCTCCTTGAAGGTG		This study; IDT
PSD1- P7_F	ATG GCT AAG GTT ATG AGG CTT ATC	Primer set used to detect PSD1 WT = 2000 bp complement = 730 bp; KO = null	This study; IDT
PSD1- P8-R	CTA TGC CAA AGC TCG ACG C		This study; IDT
PSD1-pUPRT_F	GTG CTT ACC AAT GGG GAT CTC CAG GCT AGC AAG GGC	Primer set used to amplify backbone from pUPRT-5UTR-TgNSM-TY-3UTR-CAT for the for building pPSD1-complement-CTG by Gibson assembly	This study; IDT
pUPRT-DHFR_R	GTA CAG CCT GGC GAA GCT TTG ATC TTG GAA TAG AAG GAA ACT CCA TGG		This study; IDT
pUPRT-DHFR_F	CCA TGG AGT TTC CTT CTA TTC CAA GAT CAA AGC TTC GCC AGG CTG TAC	Primer set used to amplify DHFR for the for building pPSD1-complement-CTG by Gibson assembly	This study; IDT
DHFR-PSD1_R	AAG CCT CAT AAC CTT AGC CAT CTT CCC AGA CAC GAC AAC GC		This study; IDT
DHFR-PSD1_F	GCG TTG TCG TGT CTG GGA AGA TGG CTA AGG TTA TGA GGC TT	Primer set used to generate PSD1 amplicon from T. gondii CTG strain genomic DNA	This study; IDT
PSD1-pUPRT_R	GCC CTT GCT AGC CTG GAG ATC CCC ATT GGT AAG CAC		This study; IDT

		for building pPSD1-complement-CTG by Gibson assembly	
5'Homology UPRT_F	GAG CTA CTC CTG TGT CTG AGT GG	Primer set used to linearize pPSD1-complement-CTG.	This study; IDT
3'Homology UPRT_R	GCT TAC GCA GCA GTG ACC AT		This study; IDT
UPRT-TY-CAT-Vec-MAG-F	CTGTTGTTCTTTGAAGAAATCAAGCAAGgatcttggaatagaaggaaactccatgg	Primer set used to amplify backbone from pUPRT-5UTR-TgNSM-TY-3UTR-CAT for the for building pMAG1-complement-CTG by Gibson assembly	This study; IDT
UPRT-TY-CAT-Vec-MAG-R	TCTTAGCGGAACAGGCAGCTcagGCTAGCAAGGGCTCGGG		This study; IDT
MAG-I-F	ccatggagtttccttctattccaagatcCTTGCTTGATTTCTTCAAAGAACAACAG	Primer set used to generate MAG 5UTR amplicon from T. gondii CTG strain genomic DNA for building pMAG1-complement-CTG by Gibson assembly	This study; IDT
MAG-I-R	CCCGAGCCCTTGCTAGCctgAGCTGCCTGTTCCGCTAAGA		This study; IDT
ΔMAG1- P1_F	CGTGTCGAAACAAGCTGACACTC	Primer set used to detect 5' integration of DHFR, into MAG1 locus WT =null; KO = 1400 bp	This study; IDT
ΔMAG1- P2_R	GAGACGCGTGTTCAAGCTTATCG		This study; IDT

ΔMAG1- P3_F	CGGTGGAGTCTTTCAAGCTCTC	Primer set used to detect 3’ integration of DHFR, into MAG1 locus WT =null; KO = 1200 bp	This study; IDT
ΔMAG1- P4_R	CGTGGTGTTCCTTGCCTTGATG		This study; IDT
ΔMAG1- P5_F	CCATTCAGGTGGAACTGAAC	Primer set used to detect MAG1 WT = 550 bp; KO = null	This study; IDT
ΔMAG1- P6_R	TCCTCAATCGGTGCCATATC		This study; IDT
MAG1- P7_F	ATG GAT TGC GGA CAG TGC AG	Primer set used to detect MAG1 complement = 1450 bp; KO = null	This study; IDT
MAG1-P8_R	CTG CCT GTT CCG CTA AGA TCT G		This study; IDT
ACT1 Fwd	AGAGGATACGGCTTCACCA	Primer set used to detect ACT1. 560 bp	This study; IDT
ACT1 Rev	TCGGGCAATTCATAGGACTTC		This study; IDT

Table S8 Plasmids used in this study.

Common Name	Description	Source
pUC19	Template for making gene KO constructs	
pSAG1:CAS9-GFP, U6:sgUPRT	Template for making GOI targeting Cas9 plasmids	Addgene (#54467)
pSAG1:CAS9-GFP, U6:sgMAF1	CRISPR plasmid targeting MAF1 cluster locus coding sequence for gene deletion	This study
pSAG1:CAS9-GFP, U6:sgMAG1 #1	CRISPR plasmid targeting MAG1 cluster locus coding sequence for gene deletion	(8)
pSAG1:CAS9-GFP, U6:sgPSD1	CRISPR plasmid targeting MAG1 cluster locus coding sequence for gene deletion	This study
pFloxed DHFR-TS*	Template for amplification of DHFR resistance cassette for obtaining deletion strains	This study
pMAF1locus-KO-DHFR	Plasmid containing DHFR resistance cassette flanked by by ~500bp homologous arms up and downstream of the entire MAF1 locus	This study
pMAG1KO-DHFR	Plasmid containing DHFR resistance cassette flanked by by ~500bp homologous arms up and downstream of the MAG1 locus	(8)
pPSD1-KO-DHFR	Plasmid containing DHFR resistance cassette flanked by by ~500bp homologous arms up and downstream of the MAG1 locus	This study

pUPRT-5UTR-TgNSM-TY-3UTR-CAT	Template for amplification of pUPRT and TY tag backbone for obtaining pMAG1- -Ty-complement-CAT plasmid and pPSD1-Ty-complement-CAT plasmids	This study
pMAG1-CTG -Ty-complement-CAT	*CTG-MAG1-2Ty* fusion with *CAT* drug selectable marker flanked by MAG1 5UTR and targeted to the UPRT locus. Used with pSAG1:CAS9-GFP, U6:sgUPRT to obtain MAG1 complement strain	This study
pPSD1-CTG-Ty-complement-CAT	*CTG-PSD1-2Ty* fusion with *CAT* drug selectable marker flanked by DHFR 5UTR and targeted to the UPRT locus. Used with pSAG1:CAS9-GFP, U6:sgUPRT to obtain PSD1 complement strain	This study

1. Su C, Howe DK, Dubey JP, Ajioka JW, Sibley LD. 2002. Identification of quantitative trait loci controlling acute virulence in *Toxoplasma gondii*. Proc Natl Acad Sci (USA) 99:10753-10758.
2. Huynh MH, Carruthers VB. 2009. Tagging of endogenous genes in a *Toxoplasma gondii* strain lacking Ku80. Eukaryot Cell 8:530-539.
3. Coffey MJ, Sleebs BE, Uboldi AD, Garnham A, Franco M, Marino ND, Panas MW, Ferguson DJ, Enciso M, O'Neill MT, Lopaticki S, Stewart RJ, Dewson G, Smyth GK, Smith BJ, Masters SL, Boothroyd JC, Boddey JA, Tonkin CJ. 2015. An aspartyl protease defines a novel pathway for export of Toxoplasma proteins into the host cell. Elife 4.
4. Franco M, Panas MW, Marino ND, Lee MC, Buchholz KR, Kelly FD, Bednarski JJ, Sleckman BP, Pourmand N, Boothroyd JC. 2016. A Novel Secreted Protein, MYR1, Is Central to Toxoplasma's Manipulation of Host Cells. mBio 7:e02231-15.
5. Behnke MS, Fentress SJ, Mashayekhi M, Li LX, Taylor GA, Sibley LD. 2012. The polymorphic pseudokinase ROP5 controls virulence in Toxoplasma gondii by regulating the active kinase ROP18. PLoS Pathog 8:e1002992.
6. Tobin CM, Knoll LJ. 2012. A patatin-like protein protects Toxoplasma gondii from degradation in a nitric oxide-dependent manner. Infect Immun 80:55-61.
7. Hammoudi PM, Jacot D, Mueller C, Di Cristina M, Dogga SK, Marq JB, Romano J, Tosetti N, Dubrot J, Emre Y, Lunghi M, Coppens I, Yamamoto M, Sojka D, Pino P, Soldati-Favre D. 2015.

Fundamental Roles of the Golgi-Associated Toxoplasma Aspartyl Protease, ASP5, at the Host-Parasite Interface. PLoS Pathog 11:e1005211.
8. Tomita T, Mukhopadhyay D, Han B, Yakubu R, Tu V, Mayoral J, Sugi T, Ma Y, Saeij JPJ, Weiss LM. 2021. Toxoplasma gondii Matrix Antigen 1 Is a Secreted Immunomodulatory Effector. mBio 12.
